# Bridging biosafety and biosecurity gaps: DURC and ePPP policy insights from U.S. institutions

**DOI:** 10.3389/fbioe.2024.1476527

**Published:** 2024-09-25

**Authors:** David R. Gillum, An Tran, Jennifer Fletcher, Kathleen M. Vogel

**Affiliations:** ^1^ School for the Future of Innovation and Society, Arizona State University, Tempe, AZ, United States; ^2^ Research and Innovation, University of Nevada, Reno, NV, United States; ^3^ Environmental Science and Health, University of Nevada, Reno, NV, United States; ^4^ Accountability, Assessment and Research, Chandler Unified School District, Chandler, AZ, United States

**Keywords:** dual use research of concern (DURC), enhanced potential pandemic pathogens (ePPP), biosafety, biosecurity, non-compliance reporting, risk management, biosafety survey, organizational safety

## Abstract

**Overview:**

This study provides empirical data on the knowledge and practices of biosafety and biosecurity professionals and researchers involved in research on enhanced Potential Pandemic Pathogens (ePPPs) and Dual Use Research of Concern (DURC) within various U.S. sectors. The goal is to improve public health interventions and oversight for DURC and ePPP, contributing valuable insights for policy development. A notable finding was the association between larger biosafety/biosecurity teams and a higher likelihood of conducting high-risk biological research.

**Methods:**

A survey of 541 biosafety and biosecurity professionals was conducted between March 8 and 10 April 2024, with results analyzed using SAS at a significance level of 0.05. The study received approval from the Institutional Review Boards (IRBs) at Arizona State University and the University of Nevada, Reno.

**Results:**

Government organizations were more likely to conduct DURC compared to other sectors (e.g., Academic, Commercial, Consulting). Public institutions reviewed more experiments outside the scope of the U.S. DURC Policy than private for-profit institutions. Institutions with larger biosafety/biosecurity teams reported greater research activity and more effective non-compliance reporting mechanisms (e.g., anonymous hotlines, reporting forms). Additionally, financial support and the challenges of policy implementation varied significantly across sectors.

**Discussion:**

The findings emphasize the need for appropriate staffing and resource allocation for high-risk biosafety and biosecurity research. A differentiated regulatory approach and equitable distribution of resources are essential for effective oversight. Moreover, robust non-compliance reporting systems are critical to mitigating the risks associated with DURC and ePPP research.

## 1 Introduction

Research that involves dual use research of concern (DURC) and enhanced potential pandemic pathogens (ePPP) presents unique biosafety and biosecurity hazards when compared to less risky biological research. The terminology used to describe pathogens that have the potential to cause a pandemic has evolved over time from Potential Pandemic Pathogens (PPP) to Enhanced Potential Pandemic Pathogens (ePPP) and most recently to Pathogens with Enhanced Pandemic Potential (PEPP). For clarity and consistency, this paper will use the term ePPP because it was used in the survey and is intended to encompass all three designations: PPP, ePPP, and PEPP.

DURC and ePPP experiments can benefit science, medicine, and public health but can also create risks for the deliberate or accidental release of harmful pathogens or provide knowledge, information, products or technologies that could be misapplied to do harm (Office of Science and Technology Policy, 2024). Scalable, risk-based governance that balances scientific innovation, transparency, and public trust with biosafety and biosecurity oversight is needed.

In order to better understand this issue and the discussion in this article, a few definitions are necessary:

- According to the 2014 *United States Government Policy for Institutional Oversight of Life Sciences Dual Use Research of Concern*, DURC is defined as “life sciences research that, based on current understanding, can be reasonably anticipated to provide knowledge, information, products, or technologies that could be directly misapplied to pose a significant threat with broad potential consequences to public health and safety, agricultural crops and other plants, animals, the environment, materiel, or national security” (Office of Science and Technology Policy, 2014). The policy applies to all federally funded research and requires assessments to determine if experiments involve any of the 15 listed pathogens and if it includes any of the 7 specified experiments of concern.
- According to the 2017 *United States Department of Health and Human Services Framework for Guiding Funding Decisions about Proposed Research Involving Enhanced Potential Pandemic Pathogens*, “a potential pandemic pathogen (PPP) is a pathogen that satisfies both of the following: 1) It is likely highly transmissible and likely capable of wide and uncontrollable spread in human populations; and 2) It is likely highly virulent and likely to cause significant morbidity and/or mortality in humans. An enhanced PPP is defined as a PPP resulting from the enhancement of the transmissibility and/or virulence of a pathogen. Enhanced PPPs do not include naturally occurring pathogens that are circulating in or have been recovered from nature, regardless of their pandemic potential” (U.S. Department of Health and Human Services, 2017). The policy applies to all federally funded research.
- The 2024 *United States Government Policy for Oversight of Dual Use Research of Concern and Pathogens with Enhanced Pandemic Potential*, defines biosafety as “the application of practices, controls, and containment infrastructure that reduces the risk of unintentional exposure to, contamination with, release of, or harm from pathogens, toxins, and other associated biological materials” (Office of Science and Technology Policy, 2024). The same policy defines biosecurity as “the application of security measures designed to prevent the loss, theft, misuse, diversion, unauthorized possession or material introduction, or intentional release of pathogens, toxins, biological materials, and related information and/or technology” (Office of Science and Technology Policy, 2024). The policy applies to all federally funded research.
- According to the Centers for Disease Control and Prevention (CDC) and the United States Department of Agriculture (USDA), “select agents are biological agents and toxins that have been determined to have the potential to pose a severe threat to public health and safety, to animal and plant health, or to animal or plant products.” (Centers for Disease Control and Prevention and United States Department of Agriculture, 2024; Federal Select Agent Regulations, 2002). There are 68 select agents and toxins regulated by the Federal Select Agent program (at the time of publication).

Research that involves DURC and ePPP presents unique biosafety and biosecurity hazards when compared to less risky biological research (Klotz and Sylvester, 2014; National Academies of Sciences Policy Global Affairs Committee on Science Law Committee on Dual Use Research of Concern and Options for Future Management, 2017; Evans, 2020; Shinomiya et al., 2022). DURC and ePPP experiments can benefit science, medicine, and public health but can also create risks for the deliberate or accidental release of harmful pathogens (Duprex et al., 2015; Imperiale and Casadevall, 2018; Pannu et al., 2022; Lowen et al., 2023). Scalable, risk-based governance that balances scientific innovation, transparency, and public trust with biosafety and biosecurity oversight is needed (Kanabrocki, 2011; Inglesby and Lipsitch, 2020; Evans et al., 2020; Kaplan et al., 2021; Trump et al., 2023).

The role of DURC and ePPP experiments in understanding pathogens and developing vaccines is a topic of considerable debate (Selgelid, 2009; Merler et al., 2013; Koblentz, 2014; Thevenon et al., 2015; Sandbrink et al., 2023). Researchers such as Imperiale and Casadevall (2018) and Evans et al. (2015) discuss the dual nature of this research, which, while essential for scientific advancement, poses significant biosecurity risks. The potential for accidental releases or misuse of enhanced pathogens means that stringent oversight and robust ethical frameworks are needed to ensure that the benefit of this research is not outweighed by the risks. There has been long-standing advocacy for changes in U.S. policy to enhance transparency and international cooperation in pathogen research (Lipsitch and Bloom, 2012; Casadevall and Imperiale, 2014; Schoch-Spana et al., 2017; National Academies of Sciences Policy Global Affairs Committee on Science Law Committee on Dual Use Research of Concern and Options for Future Management, 2017; Gupta, 2023). Experts emphasize the need for improved frameworks to balance the benefits and risks of DURC and ePPP research (National Academies of Sciences Engineering and Medicine, 2016; Parker, 2023). These recommendations call for creating a research environment that ensures safety without hindering innovation.

Debates surrounding dual-use biological research have been ongoing since at least 2001. Although the 2004 National Research Council report, “Biotechnology Research in an Age of Terrorism,” often serves as a key reference point, earlier discussions had already begun to address these issues (National Research Council Policy Global Affairs, 2004). Notable examples include Rachel Nowak’s 2001 article on the killer mousepox virus (Nowak, 2001), Gerald Epstein’s 2001 analysis on controlling biological warfare threats (Epstein, 2001), and subsequent works by scholars like Kathryn Nixdorff and Wolfgang Bender (Nixdorff and Bender, 2002), and George Poste (Poste, 2002), among others (Zilinskas and Tucker, 2002; Harris and Steinbruner, 2003; Kellman, 2003). These discussions laid the groundwork for the development of biosafety and biosecurity policies aimed at governing DURC and ePPP.

Since then, three important biosafety and biosecurity policies were created in an attempt to govern DURC and ePPP experiments: the *2014 United States Government Policy for Institutional Oversight of Life Sciences Dual Use Research of Concern,* the 2017 *Framework for Guiding Funding Decisions about Proposed Research Involving Enhanced Potential Pandemic Pathogens,* and the 2017 *Recommended Policy Guidance for Departmental Development of Review Mechanisms for Potential Pandemic Pathogen Care and Oversight (P3CO)* (Office of Science and Technology Policy, 2014; Office of Science and Technology Policy, 2017; U.S. Department of Health and Human Services, 2017). The 2014 DURC policy established guidelines for identifying, reviewing, and mitigating risks associated with life sciences research that could be misused for malevolent ends. The 2017 ePPP funding policies provided a risk-based approach for agencies to assess and oversee research involving ePPPs. This survey was conducted prior to the new DURC/PEPP policy issued in May 2024; therefore, respondents were commenting on the 2014 DURC and 2017 P3CO policies.

However, this research shows that there remain considerable differences in how institutions interpret, implement, and comply with these policies. Factors such as institutional sector (e.g., public, private), size (e.g., large, small), available resources (e.g., amount of funding, size of biosafety team), and organizational category (i.e., academic, commercial, consulting, government, other) can influence the quality and effectiveness of biosafety and biosecurity at an institution (U.S. Department of Health and Human Services, 2014; U.S. Government Accountability Office, 2023). A biosafety team, which is responsible for ensuring the safe handling and containment of biological agents, is what Huising and Silbey might consider an *accountability infrastructure*, or “a network of offices, roles, programs, and procedures dedicated to aligning the organization’s operations with external standards, codes of conduct, ethical and normative expectations, and regulations” (Huising and Silbey, 2021, S40). The presence of effective accountability infrastructures is crucial for maintaining rigorous safety and compliance standards. This research is the first study to examine how the accountability infrastructures for biosafety in institutions can affect DURC and ePPP implementation.

This study can help inform future applied biosafety and biosecurity research and implementation regarding DURC and ePPP experiments. For example, it could also be useful in developing interview questions and creating typologies to assess biosafety and biosecurity compliance for DURC and ePPP activities. Typologies, which are systematic classifications based on shared characteristics, can help categorize different practices and policies, making it easier to compare and analyze how various institutions manage biosafety and biosecurity (McNie et al., 2015; 2016). These typologies include factors such as organizational characteristics, compliance conditions, and research gaps, providing a multi-dimensional framework to understand the complexities of managing DURC and ePPP. By examining these factors, institutions can develop more effective strategies to ensure comprehensive risk management and compliance mechanisms. Understanding these typologies will guide future research and policy development, ensuring that biosafety and biosecurity policies evolve to meet the challenges posed by advanced biotechnology.

## 2 Methods

To better understand how individuals and institutions manage these biosafety and biosecurity risks, we conducted a baseline assessment on the state of DURC and ePPP biosafety and biosecurity knowledge to proactively identify strengths, weaknesses, and areas for improvement in future policy development. Data were collected through surveys administered to individuals affiliated with the American Biological Safety Association International (ABSA International) and those listed as Institutional Biosafety Committee contacts with the National Institutes of Health Office of Science Policy in 2024 in alignment with prior research of this profession (Fletcher et al., 2021; Gillum et al., 2013; Gillum et al., 2014; Gillum et al., 2016). 1096 individuals received the survey, and 541 respondents completed the survey in full or partial response, representing approximately 49.4% of the total recipients. Of these respondents, 21 were from countries outside of the United States and were eliminated from the study, as our grant was specific to only those located inside the United States. Participation in the survey was optional and voluntary. Because respondents were not required to complete every question, and respondents could skip or select “Prefer not to say” for certain questions, the total number of responses for each question varied. All survey questions and possible responses may be found in Supplementary Material SA. Questions regarding perceived *effectiveness* were measured on a Likert Scale, with 5 = Extremely effective, 1 = Not effective at all. Questions regarding perceived *financial support* were measured as 5 = Excellent and 1 = Poor. Questions regarding *difficulty* were measured as 5 = Extremely difficult and 1 = Extremely easy. Questions regarding perceived *impact* were measured as 5 = Extremely positive and 1 = Extremely negative. Analyses were conducted using SAS. All research complied with federal guidelines and institutional policies related to human subjects research. This project was funded by the National Institutes of Health’s (NIH) National Institute of General Medical Sciences (NIGMS) award #1R01GM155913-01 and was approved by the Institutional Review Boards at Arizona State University (IRB# 00016457) and the University of Nevada, Reno (IRB # 2102871).

### 2.1 Respondents

The survey had 520 respondents who worked in the United States. Out of these respondents, 117 (22.5%) identified as female, 127 (24.4%) as male, and 276 as other or did not specify their gender. The age distribution of the respondents showed that 3 (0.58%) were between 21 and 30 years, 40 (7.69%) were between 31 and 40 years, 83 (15.96%) were between 41 and 50 years, 68 (13.08%) were between 51 and 60 years, 43 (8.27%) were between 61 and 70 years, 9 (1.73%) were between 71 and 80 years, 1 (0.19%) was 81 years or more, and 273 (52.5%) did not specify their age. The racial distribution of respondents was as follows: 210 (40.38%) identified as White, 13 (2.50%) as Asian, 7 (1.35%) as Black or African American, 1 (0.19%) as American Indian or Alaska Native, 2 (0.38%) as Native Hawaiian or Pacific Islander, and 287 (55.19%) as other, preferred not to say, or did not specify their race.

Regarding educational background, 1 (0.19%) respondent was a high school graduate or equivalent, 40 (7.69%) had a bachelor’s degree, 81 (15.58%) had a master’s degree, 123 (23.65%) had a doctorate and 275 (52.88%) did not specify their educational level. For details on respondent demographics, see Table 1.

**TABLE 1:** Respondents demographic data.

Question	Response	Number	Percentage
What is your gender?	Female	117	22.5
	Male	127	24.42
	Other/Not Specified	276	53.08
What is your age range?	21–30	3	0.58
	31–40	40	7.69
	41–50	83	15.96
	51–60	68	13.08
	61–70	43	8.27
	71–80	9	1.73
	81 or more	1	0.19
	No specified	273	52.5
What is your highest education level?	High school graduate or equivalent	1	0.19
	Bachelor’s degree or equivalent	40	7.69
	Master’s degree or equivalent	81	15.58
	Doctorate	123	23.65
	Other/Not specified	275	52.88
What is your total accumulated years of experience performing biosafety/biosecurity - related duties?	Less than 1 year	3	0.58
	At least 1 year but less than 5 years	32	6.15
	At least 5 years but less than 10 years	46	8.85
	At least 10 years but less than 15 years	57	10.96
	At least 15 years but less than 20 years	41	7.88
	More than 20 years	63	12.12
	Other/No specified	278	53.46
What is your salary range in US dollars?	<$50,000	2	0.38
	>$50,000-$100,000	89	17.12
	>$100,000-$150,000	68	13.08
	>$150,000-$200,000	39	7.5
	>$200,000	18	3.46
	Not specified	304	58.46
What is your race?	White	210	40.38
	Asian	13	2.5
	Black or African American	7	1.35
	American Indian or Alaska Native	1	0.19
	Native Hawaiian or Pacific Islander	2	0.38
	Other/Prefer not to say/No specified	287	55.19
What is your ethnicity	White	210	40.38
	Asian	13	2.5
	Black or African American	7	1.35
	Native Hawaiian or Pacific Islander	2	0.38
	American Indian or Alaska Native	1	0.19
	Not specified	281	55
What is your sexual orientation	Heterosexual	208	40
	Homosexual	12	2.31
	Bisexual	9	1.73
	Other	3	0.58
	Not specified	288	55.38

Out of 445 respondents who answered questions about their roles at their institutions, 306 said that their position includes biosafety and biosecurity responsibilities, while 92 individuals said they only practiced biosafety but not biosecurity. Conversely, 14 individuals practiced only biosecurity and no biosafety. Approximately 70% of respondents reported that they spent between 75% and 100% of their time on biosafety responsibilities. About 7% of respondents said that they spent between 75% and 100% of their time on biosecurity responsibilities. For details on respondents’ workplace and their position characteristics, see Table 2.

**TABLE 2:** Workplace factors data.

Question	Response	Number	Percentage
Does your workplace receive funding from the U.S. Government?	Yes	368	70.77
	No	67	12.88
	I do not know	15	2.88
	Not specified/NA	70	13.46
Which category best describes the institution where you work?	R1 Doctoral University - Very High Research Activity	171	32.88
	R2 Doctoral University - High Research Activity	48	9.23
	Master’s College or University (Carnegie M1-M3)	8	1.54
	Undergraduate University/Primary Teaching Institution	18	3.46
	Government - Federal, State, City, Tribal	65	12.5
	Government Owned/Privately Operated	7	1.35
	Consulting Company	8	1.54
	Contract Research Organization	14	2.69
	Commercial	11	2.12
	Pharmaceutical	15	2.88
	Clinical or Diagnostic Testing	22	4.23
	Other	71	13.65
How would you characterize the institution where you work?	Public	257	49.42
	Private - Non-Profit	111	21.35
	Private - For-Profit	74	14.23
	I do not know	12	2.31
	Not specified/NA	66	12.69
How many biosafety/biosecurity practitioners work at your institution?	None	19	3.65
	1	68	13.08
	2	81	15.58
	3	45	8.65
	4	42	8.08
	5 or more	137	26.35
	I do not know	36	6.92
	Not Specified/NA	92	17.69
Are you currently employed in a position with biosafety responsibilities?	Yes	418	80.38
	No	71	13.65
	Not specified/NA	31	5.96
Are you in a role with biosecurity responsibilities?	Yes	321	61.73
	No	127	24.42
	Not specified/NA	72	13.85
Does your workplace conduct research that is subject to the U.S. Government Policy on Dual Use Research of Concern?	Yes	126	24.23
	No	231	44.42
	I do not know	50	9.62
	Not specified/NA	113	21.73
Does your workplace conduct research that may generate enhanced Potential Pandemic Pathogens (ePPP)?	Yes	63	12.12
	No	254	48.85
	I do not know	44	8.46
	Not specified/NA	159	30.58
Approximately how much of your time is spent on biosafety and how much is spent on biosecurity?	Primarily biosafety (∼100% biosafety/∼0% biosecurity)	45	8.65
	Mostly biosafety with a little biosecurity (∼75% biosafety/∼25% biosecurity)	160	30.77
	About the same amount of biosafety and biosecurity (∼50% biosafety/∼50% biosecurity)	73	14.04
	Mostly biosecurity with a little biosafety (∼75% biosecurity/∼25% biosafety)	12	2.31
	Primarily biosecurity (∼100% biosecurity/∼0% biosafety)	5	0.96
	Not Specified/NA	225	43.27

## 3 Results

Prior analyses on differences in institutional practices within the biosafety and biosecurity community (Fletcher et al., 2021; Gillum et al., 2013; Gillum et al., 2014; Gillum et al., 2016) have been conducted. In following the analytic approach in these studies, we aim to study the differences in institutional practices related to DURC and ePPP across different types and categories of institutions. Respondents had the option to select from a variety of choices for institution type. However, due to the small number of respondents from certain categories of institutions, they were grouped according to industry type, operational function, and prior research, resulting in the following list referred to as *Organizational Categories*: Academic [(Carnegie R1 - Very High Research Doctoral University and Carnegie R2 - High Research University, Undergraduate University/Primary Teaching Institution and Master’s College or University (Carnegie M1-M3)]; Government (Government-Federal, State, City, Tribal and Government Owned/Privately Operated); Consulting (Consulting Company and Contract Research Organization), Commercial (Commercial, Pharmaceutical and Private For-Profit Clinical or Diagnostic Testing) and Other (Other, Public and Private-Non Profit Clinical or Diagnostic Testing). Institutions were also categorized as *Sectors:* Public, Private For-Profit and Private Non-Profit as selected by the respondent. The number of biosafety/biosecurity practitioners working at the institution was another characteristic to categorize the institutions. Chi-square, Fisher’s exact test (used as a replacement for the Chi-square test when the expected frequency of one or more cells is less than five) and ANOVA were used to evaluate differences. A significance level of 0.05 was used to determine statistical significance.

### 3.1 Dual use research of concern (DURC) results

Statistical analyses were done on whether or how institutions conducted or reviewed research subject to the 2014 *United States Government Policy for Institutional Oversight of Life Sciences Dual Use Research of Concern*. Statistically significant differences were found across Organizational Categories [χ^2^(4) = 15.80, *p* < .05]. A larger proportion of respondents who work at Government (28 out of 54, 51.85%) institutions indicated that they conduct what they considered to be DURC compared to other Organizational Categories while Commercial had the lowest proportion (5 out of 29, 17.24%). Likewise, Public institutions are more likely to conduct DURC with 42.58% (89 out of 209) compared to 18.37% (9 out of 49) in Private For-Profit and 27.11% (25 out of 92) in Private Non-Profit [χ^2^(2) = 13.69, *p < .05*]. There was also a positive correlation between the size of an institution’s biosafety/biosecurity team and the likelihood of conducting research that is subject to DURC policy [χ^2^(4) = 47.64, *p* < 0.001]. The outsourcing of DURC (i.e., the practice of contracting or delegating DURC to another institution) showed no significant differences across Organizational Categories (*p* = 0.15) and across Public/Private Sectors (*p* = 0.17) (see Table 3 for details).

**TABLE 3:** Dual use research of concern (DURC) results.

Simplified questions from Supplementary Material SA	Group	Workplace conducts research that is subject to DURC (question #18)		Chi square test	Workplace outsources DURC (question #19)		Fisher’s exact test	Workplace conducts reviews for DURC for experiments that are not covered by US DURC (question #21)		Chi square test
		Yes (%)	No (%)		Yes (%)	No (%)		Yes (%)	No (%)	
Organizational categories	Academic	37.68	62.32	15.80*	7.45	92.55	0.00	51.67	48.33	13.26*
	Commercial	17.24	82.76		7.41	92.59		28.00	72.00	
	Consulting	27.27	72.73		0.00	100.00		60.00	40.00	
	Government	52.85	48.15		19.15	80.85		40.00	60.00	
	Other	21.82	78.18		6.12	93.88		28.00	72.00	
Sector	Public	42.58	57.42	13.69*	88.41	11.59	0.01	45.78	54.22	7.18*
	Private- for-profit	18.37	81.63		96.00	4.00		27.27	72.73	
	Private-non-profit	27.17	72.83		94.59	5.41		51.81	48.19	
Biosafety/Biosecurity team size	1	14.29	85.71	47.64***	0	100	0.07	20	80	22.96*
	2	17.14	82.86		8.2	91.8		45	55	
	3	21.05	78.95		2.94	97.06		48.57	51.43	
	4	42.5	57.5		6.67	93.33		65.71	34.29	
	5 or more	55.93	44.07		12.5	87.5		56.38	43.62	

Notes. *p < .05, **p < .01, ***p < .001.

Under the 2014 *United States Government Policy for Institutional Oversight of Life Sciences Dual Use Research of Concern*, assessments are required to determine if the research involves any of the 15 listed pathogens and if it includes any of the 7 specified experiments of concern (U.S. Department of Health and Human Services, 2014). Thus, a significant finding was related to who was conducting reviews for DURC [χ^2^(4) = 13.26, *p* < .05] above and beyond the requirements of the policy (e.g., conducting biosafety and biosecurity assessments for more than the 15 agents and/or 7 experimental categories). Results showed that Consulting institutions reported the highest proportion of reviews for DURC in experiments that are not covered by the 2014 DURC policy (6 out of 10, 60%), followed by Academic institutions (93 out of 180, 51.67%) and Government entities (14 out of 35, 40%), whereas Commercial and Other sectors both reported the lowest rate (7 out of 25 and 14 out of 50 respectively, 28%).

Public (76 out of 166, 45.78%) and Private Non-Profit (43 out of 83, 51.81%) institutions are more likely to review experiments beyond the DURC policy compared to Private For-Profit institutions (12 out of 44, 27.27%) [χ^2^(4) = 7.18, *p* < .05]. Additionally, there was a significant difference based on the size of an institution’s biosafety/biosecurity team. Institutions with a team of four professionals reported the highest proportion of reviews beyond the DURC policy (10 out of 50, 65.71%), followed by teams of 5 or more (53 out of 94, 56.38%%), teams of 3 (17 out of 35, 48.57%), teams of 2 (27 out of 60, 45%), and single-person teams (10 out of 50, 20%) [χ2(4) = 22.96, *p* < .05].

### 3.2 Enhanced potential pandemic pathogen (ePPP) results

Analyses regarding research that could produce ePPP showed predominantly non-significant results (see Table 4 for details), with no statistically significant differences observed across Organizational Categories or Sectors. However, a notable finding was the relationship between the size of the biosafety/biosecurity team and the likelihood of an institution conducting research that could generate ePPP, indicating that larger teams may be more involved in such research. The number of biosafety/biosecurity staff is positively correlated with the performance of ePPP [χ^2^(4) = 16.86, *p* < .05]. For outsourcing research subject to the 2017 *Framework for Guiding Funding Decisions about Proposed Research Involving Enhanced Potential Pandemic Pathogens*, no significant differences were observed across Organizational Categories and Sectors.

**TABLE 4:** Enhanced potential pandemic pathogen (ePPP) results.

Simplified questions from Supplementary Material SA	Group	Workplace conducts ePPP (question #32)		Chi-square test	Workplace outsources ePPP (question #33)		Fisher’s exact test	Workplace reviews research under ePPP (question #36)		Chi-square test	Fisher’s exact test
		Yes (%)	No (%)		Yes (%)	No (%)		No (%)	Yes (%)		
Organizational Categories	Academic	23.2	76.8	3.95	2.53	97.47	0.01	85.82	14.18		0.00
	Commercial	22.73	77.27		4.17	95.83		85.71	14.29		
	Consulting	9.09	90.91		0.00	100.00		100.00	15.38		
	Government	15.91	84.09		8.11	91.89		85.62	15.38		
	Other	13.79	86.21		2.04	97.96		93.33	6.67		
Sector	Public	20.79	79.21	0.42	3.77	96.23	0.06	86.15	13.85	0.39	
	Private- for-profit	17.78	82.22		4.88	95.12		88.89	11.11		
	Private-non-profit	17.86	82.14		1.37	98.63		88.73	11.27		
Biosafety/Biosecurity team size	1	4.26	95.74	16.86*	0	100	0.62	7.32	92.68		0.14
	2	16.92	93.08		3.57	96.43		10	90		
	3	13.89	86.11		3.03	96.97		9.68	90.32		
	4	24.24	75.76		0	100		8.33	91.67		
	5 or more	31.63	68.37		4.76	95.24		22.86	77.14		

Notes. *p < .05, **p < .01, ***p < .001.

The review of research under the U.S. ePPP Policy also showed no significant differences among Organizational Categories. Similarly, there was no significant difference observed in the review of ePPP research in different Sectors and the biosafety/biosecurity team. This may be attributed to the fact that fewer individuals reported ePPP research at their institution as compared to FSAP and DURC.

### 3.3 Perception of financial support, difficulties and impact

Analyses were conducted on questions surrounding financial support, difficulties in implementing the policies, and impact of managing DURC, ePPP experiments, and Federal Select Agent Program (FSAP) activities across Organizational Categories and Sectors (see Table 5). The analyses also considered the effects of managing these policies within Organizational Categories and Sectors (see Table 6). DURC, ePPP, and FSAP each focus on different aspects of biosafety and biosecurity related to biological research, but they have distinct objectives and regulatory frameworks. FSAP oversees the possession, use, and transfer of select agents and toxins that have the potential to pose a severe threat to public, animal, or plant health, or to animal or plant products and is jointly managed by the Centers for Disease Control and Prevention (CDC) and the Animal and Plant Health Inspection Service (APHIS) of the U.S. Department of Agriculture (USDA). In contrast, DURC and ePPP are U.S. Government funding policies specifically aimed at overseeing research that could potentially be misused to pose significant threats. Understanding these distinct frameworks and their implications is essential for evaluating the comprehensive management of biosafety and biosecurity practices across different types of institutions.

**TABLE 5:** Differences in perception of financial support, difficulties and impact across organizational categories and sectors.

	Academic M (SD)	Commercial M (SD)	Consulting M (SD)	Government M (SD)	Other M (SD)	df^	Levene	F	ηp2	Public M (SD)	Private-for-profit M (SD)	Private-non profit M (SD)	df^	Levene	F	ηp2
Amount of financial support provided for DURC oversight (Question #27)	2.13 (±1.21)	2.25 (±±.04)	2.67 (±1.37)	2.77 (±1.33)	2.00 (±0.97)	186	0.86	2.06	0.04	2.42^a^ (±1.28)	2.08 (±1.16)	1.89^a^ (±1.03)	181	2.12	3.65*	0.04
Amount of financial support provided for ePPP oversight (Question 42)	1.83 (±1.12)	1.67 (±1.03)	3.00 (±1.22)	2.2 (±0.98)	2.12 (±0.99)	155	0.28	2.01	0.05	2.00 (±1.12)	1.85 (±1.05)	1.83 (±1.27)<	154	0.25	0.35	0.00
Amount of financial support provided for FSAP oversight (Question #50)	2.87 (±1.22)	2.67 (±0.58)	3.40 (±0.94)	2.96 (±0.87)	3.10 (±1.19)	151	2.20	0.37	0.01	3.00 (±1.15)	2.88 (±1.25)	2.64 (±1.11)	150	0.08	1.28	0.00
Difficulty of administering DURC policy (Question #28)	2.96 (±1.01)	2.80 (±1.55)	3.00 (±1.26)	2.61 (±1.09)	2.48 (±0.85)	231	2.44*	1.76	0.03	2.85 (±0.98)	2.76 (±1.44)	2.93 (±1.05)	225	4.25*	0.2	0.00
Difficulty of administering ePPP policy (Question #43)	3.23 (±0.87)	3.33 (±1.03)	2.80 (±0.84)	2.93 (±0.96)	2.8 (±0.65)	166	0.75	1.83	0.04	3.14 (±0.85)	3.21 (±0.86)	2.77 (±1.01)	164	0.27	1.35	0.02
Difficulty of administering FSAP policy (Question #51)	3.10 (±0.99)	3.40 (±1.14)	3.60 (±0.55)	2.86 (±0.91)	2.27 (±0.90)	153	0.69	2.58*	0.06	3.09 (±0.99)	2.89 (±1.17)	2.91 (±0.98)	152	0.2	0.53	0.01
Impact of DURC on research (Question #30)	3.00^a^ (±0.61)	3.42 (±0.9)	3.33 (±0.92)	3.57^a^ (±0.98)	3.22 (±0.64)	238	3.45**	5.46***	0.09	3.16 (±0.73)	3.40 (±0.75)	3.00 (±0.70)	233	0.05	2.49	0.02
Impact of ePPP on research (Question #45)	2.97 (±0.63)	3.33 (±0.87)	3.50 (±0.84)	3.27 (±0.89)	3.26 (±0.69)	175	0.88	2.43	0.05	3.02^a^ (±0.69)	3.58^ab^ (±0.77)	2.98^b^ (±0.63)	174	0.31	5.88*	0.06
Impact of FSAP on research (Question #53)	3.14 (±0.85)	3.40 (±0.7)	3.57 (±0.79)	3.10 (±0.66)	3.20 (±0.71)	221	0.90	0.75	0.01	3.13 (±0.84)	3.43 (±0.68)	3.20 (±0.76)	217	0.91	1.32	0.01

Notes. *p < .05, **p < .01, ***p < .001. Groups sharing the same symbol (a, b) are significantly different from each other at the *0.05* significance level according to the Tukey HSD, test. ^ Degree Freedom (*df*) is the same for Levene’s test and F test.

**TABLE 6:** Differences in the effects of managing policies across organizational categories and sectors.

		Financial support							Difficulty							Impact						
		DURC	ePPP	FSAP	df^	Levene	F	ηp2	DURC	ePPP	FSAP	df^	Levene	F	ηp2	DURC	ePPP	FSAP	df^	Levene	F	ηp2
		M (SD)	M (SD)	M (SD)					M (SD)	M (SD)	M (SD)					M (SD)	M (SD)	M (SD)				
Organizational Categories	Academic	2.13^a^ (±1.21)	1.83^b^ (±1.12)	2.87^ab^ (±1.22)	338	0.55	21.90***	0.12	2.96 (±1.01)	3.23 (±0.87)	3.10 (±0.99)	385	1.82	2.65	0.01	3.00 (±0.61)	2.97 (±0.63)	3.14 (±0.85)	428	7.33***	2.30	0.01
	Commercial	2.25 (±±.04)	1.67 (±1.03)	2.67 (±0.58)	16	0.81	1.17	0.14	2.8 0 (±1.55)	3.33 (±1.03)	3.40 (±1.14)	20	1.5	0.47	0.05	3.42 (±0.9)	3.33 (±0.87)	3.40 (±0.7)	30	0.32	0.03	0.00
	Consulting	2.67 (±1.37)	3.00 (±1.22)	3.40 (±0.94)	15	0.41	0.51	0.07	3.00 (±1.26)	2.8 (±0.84)	3.60 (±0.55)	15	2.14	0.95	0.13	3.33 (±0.92)	3.50 (±0.84)	3.57 (±0.79)	18	0.01	0.14	0.02
	Government	2.77 (±1.33)	2.20 (±0.98)	2.96 (±0.87)	74	4.63*	2.66	0.07	2.61 (±1.09)	2.93 (±0.96)	2.86 (±0.91)	66	0.55	0.65	0.02	3.57 (±0.98)	3.27 (±0.89)	3.10 (±0.66)	82	1.80	2.49	0.06
	Other	2.00^a^ (±0.97)	2.12 (±0.99)	3.10^a^ (±1.19)	47	0.49	4.04*	0.15	2.48 (±0.85)	2.80 (±0.65)	2.27 (±0.90)	62	0.93	2.04	0.06	3.22 (±0.64)	3.26 (±0.69)	3.20 (±0.71)	74	0.07	0.05	0.00
Sector	Public	2.42^ab^ (±1.28)	2.00^ac^ (±1.12)	3.00^bc^ (±1.15)	323	1.8	18.54***	0.10	2.85 (±0.98)	3.14 (±0.85)	3.09 (±0.99)	349	0.16	3.31*	0.02	3.16 (±0.73)	3.02 (±0.69)	3.13 (±0.84)	400	1.96	0.95	0.00
	Private-Profit	2.08 (±1.16)	1.85 (±1.05)	2.88 (±1.25)	31	0.07	1.81	0.11	2.76 (±1.44)	3.21 (±0.86)	2.89 (±1.17)	38	1.54	0.03	0.00	3.4 (±0.75)	3.58 (±0.77)	3.43 (±0.68)	59	0.17	0.33	0.01
	Private-NoProfit	1.89^a^ (±1.03)	1.83^b^ (±1.27)	2.64^ab^ (±1.11)	131	0.08	6.51**	0.09	2.93 (±1.05	2.77 (±1.01)	2.91 (±0.98)	154	1.17	1.51	0.02	3.00 (±0.70)	2.98 (±0.63)	3.20 (±0.76)	165	0.44	1.65	0.02

Notes. *p < .05, **p < .01, ***p < .001. Groups sharing the same symbol (a, b, c) are significantly different from each other at the *0.05* significance level according to the Tukey HSD, test. ^ Degree Freedom (*df*) is the same for Levene’s test and F test.

The perceived impact of the DURC policy on research ranged from “extremely negative” to “extremely positive” among organization types [*F*(4) = 5.46, *p* < 0.001, *np* ^*2*^ = 0.09]. The Tukey *post hoc* tests revealed significant differences between Academic (*M* = 3.00, *SD* = 0.61) and Government (*M* = 3.57, *SD* = 0.98) Organizational Categories. A significant difference was also noted between Public, Private For-Profit, and Private Non-Profit and the amount of financial support for DURC oversight [*F*(2) = 3.65, *p* < .05, *np* ^*2*^ = 0.04]. Tukey *post hoc* tests also revealed the differences were found between Public (*M* = 2.42, *SD* = 1.28) and Private Non-Profit (*M* = 1.89, *SD* = 1.03) institutions.

For DURC oversight, responses to survey question #27, which asked respondents to rate the adequacy of financial support from “Poor” to “Excellent,” did not reveal significant differences in perceptions among various Organizational Categories. Additionally, the difficulty in managing the DURC policy (e.g., easy, difficult) did not significantly differ across Organizational Categories or Sectors. Moreover, statistically significant differences were not found between Sectors and perceived impact of the DURC policy.

For ePPP oversight, the impact of the U.S. ePPP Policy on research was perceived differently among sectors [*F(2)* = 5.88, *p* < 0.001, *np* ^*2*^ = 0.06]. Tukey *post hoc* tests revealed that the impact of the ePPP policy on Private For-Profit (*M* = 3.58, *SD* = 0.77) differed from both Public (M = 3.02, SD = 0.69) and Private Non-Profit (*M* = 2.98, *SD* = 0.63). All other findings were not significant: perceived financial support across Organizational Categories or Sectors, and the difficulty in managing ePPP policy among Organizational Categories or Sectors.

Regarding FSAP oversight, the difficulty of managing FSAP regulations did differ significantly among Organizational Categories [*F(4)* = 2.58, *p* < 0.05, *np* ^*2*^ = 0.06] with the highest difficulty perceived in Consulting (*M* = 3.60, *SD* = 0.79), followed by Commercial (*M* = 3.40, *SD* = 0.70), Academic (*M* = 3.10, *SD* = 0.99), Government (*M* = 2.86, *SD* = 0.91) and Other (*M* = 2.27, *SD* = 0.90). This finding suggests that certain characteristics relevant to managing FSAP regulations, such as the number of FSAP projects or differences in institutional management practices, may vary across these categories. Understanding these differences could provide insights into why these variations in perceived difficulty exist and whether they have practical implications. Tukey *post hoc* test did not reveal significant differences among the groups. All other findings were not significant: financial support amongst Organizational Categories or Sectors, the difficulty of managing the same policy among Sectors, and the impact of FSAP regulations across Organizational Categories or Sectors.

While the previous section compared the financial support, difficulties in managing each policy, and impacts of each policy individually in the various Organizational Categories and Sectors, the purpose of the following analysis is to compare the financial support within Organizational Categories and Sectors for all of the policies. This approach will examine how financial support, perceived difficulty in managing, and impact vary among all of the policies.

For those in the Academic category, there were clear differences in the perceived adequacy of financial support for each policy. FSAP oversight was perceived as receiving the most support, with an average score of 2.87 out of 5 (*SD* = 1.22) on a scale from “Poor” to “Excellent.” In contrast, DURC and ePPP oversight were perceived as receiving less support, with scores of 2.13 (*SD* = 1.21) and 1.83 (*SD* = 1.12), respectively. It is important to note that FSAP is mandated by law, which may influence these perceptions. ANOVA tests showed that these differences in funding levels were significant [*F(2)* = 21.9, *p* < 0.001, *np* ^*2*^ = 0.12]. Similarly, the Other category showed significant differences [*F(2)* = 4.04, *p* < 0.05, *np* ^*2*^ = 0.15], with the Tukey *post hoc* test revealing the difference between financial support for FSAP (*M* = 3.10, *SD* = 1.19) and DURC (*M* = 2.00, *SD* = 0.97). These differences suggest that biosafety officers may not be able to allocate as much attention to DURC and ePPP oversight due to limited resources, potentially leading to gaps in oversight. The day-to-day implications could include increased strain on biosafety and biosecurity professionals, who may already be stretched thin by the stringent legal requirements of FSAP. As new policies in life sciences continue to emerge, this burden could grow, raising concerns about the sustainability of effective oversight. If all these policies were to become law, the pressure on these practitioners could become overwhelming, highlighting the need for adequate and balanced financial support across all areas of biosafety and biosecurity.

The Public Sector showed noticeable differences as well [*F(2)* = 18.54, *p* < 0.001, *np* ^*2*^ = 0.10]. FSAP had a financial support score of 3.00 (*SD* = 1.15), DURC had 2.42 (*SD* = 1.28), and ePPP had 2.00 (*SD* = 1.12). FSAP received significantly more funding than the other two policies. DURC received more support than ePPP. In the Private Non-Profit sector there were also significant differences [*F(2)* = 6.51, *p* < 0.01, *np* ^*2*^ = 0.09]. FSAP again received the most financial support, with a score of 2.63 (*SD* = 1.11). DURC and ePPP were funded less, with scores of 1.89 (*SD* = 1.03) and 1.83 (*SD* = 1.27) respectively.

The study also looked at how difficult these policies were to manage in the workplace, with significant differences noted in the Public sector [*F(2)* = 3.31, *p* < .05, *np* ^*2*^ = 0.02]. The ePPP policy was perceived as the most challenging to manage, with a score of 3.14 (*SD* = 0.85). FSAP was close behind with 3.09 (*SD* = 0.99), and DURC was seen as the least difficult, scoring 2.85 (*SD* = 0.99). While there were differences in how difficult each policy was perceived to be, they were not hugely significant according to Tukey *post hoc* tests.

### 3.4 Effectiveness of risk reduction

The study looked at how well different methods for reducing risks were working, especially focusing on how institutions report non-compliance, like using anonymous whistleblower hotlines or online forms. Results showed that the perceived effectiveness (e.g., effective, not effective) of these reporting methods varied significantly between different types of Organizational Categories [*F(4)* = 2.78, *p <* 0.05]. The Tukey *post hoc* test revealed that Commercial organizations rated their reporting effectiveness at 3.35 out of 5 (*SD* = 1.18), which was significantly lower than Government organizations, which rated theirs at 4.05 out of 5 (*SD* = 0.84). See Table 7 for more details.

**TABLE 7:** Differences in effectiveness of risk reduction across organizational categories and sectors.

Simplified questions from Supplementary Material SA	Group	Effectiveness of workplace’s non-compliance reporting mechanism (question #16)	df	Levene	F	ηp2	Effectiveness of DURC policy in reducing risks (question #55)	df^	Levene	F	ηp2	Effectiveness of ePPP policy in reducing risks (question #57)	df^	Levene	F	ηp2
		M (SD)					M (SD)					M (SD)				
Organizational Categories	Academic	3.72 (±0.93)	259	1.13	2.78*	0.04	2.78 (±0.93)	242	1.55	0.42	0.08	2.62 (±0.94)	194	1.20	0.61	0.00
	Commercial	3.35 (±1.18)					2.92 (±1.24)					2.40 (±1.26)				
	Consulting	4.33 (±0.82)					2.71 (±1.14)					2.55 (±1.04)				
	Government	4.05 (±0.84)					2.97 (±1.05)					2.74 (±0.94)				
	Other	3.63 (±0.88)					2.70 (±0.78)					2.85 (±0.82)				
Sector	Public	3.82 (±0.88)	254	1.25	1.95	0.01	2.86 (±0.94)	235	0.05	1.24	0.01	2.71 (±0.90)	189	0.41	1.17	0.00
	Private- For-Profit	3.55 (±1.06)					2.76 (±0.99)					2.69 (±1.01)				
	Private-Non Profit	3.59 (±1.00)					2.63 (±0.94)					2.47 (±0.97)				
Biosafety/Biosecurity Team Size	1	3.32 (±1.08)	238	1.14	5.29**	0.08	2.71 (±1.08)	220	1.42	1.4	0.03	2.72 (±1.03)	175	0.94	1.82	0.04
	2	3.42 (±0.84)					2.82 (±0.97)					2.68 (±1.02)				
	3	3.7 (±0.1)					2.93 (±0.73)					2.76 (±0.77)				
	4	3.77 (±0.88)					2.39 (±1.07)					2.13 (±0.92)				
	5 or more	3.99 (±0.84)					2.81 (±0.9)					2.63 (±0.89)				

Notes. ^ Degree Freedom (*df*) is the same for Levene’s test and F test.

Institutions with larger biosafety and biosecurity teams were significantly [*F(4)* = 5.29, *p* < 0.001] better at identifying potential issues with risky experiments (like DURC and ePPP experiments). The Tukey *post hoc* test showed that teams with 5 or more people had an effectiveness rating of 3.99 (*SD* = 0.84), which was significantly higher compared to teams with only 1 or 2 people, who rated their effectiveness at 3.32 (*SD* = 1.08) and 3.42 (*SD* = 0.84), respectively (see Table 7).

The study also looked at the perceived effectiveness for how the U.S. policies (DURC and ePPP) were at reducing risks. On average, the DURC policy was rated 2.80 out of 5 and the ePPP policy was rated 2.65 out of 5. However, there were no significant differences in these ratings across different types of Organizational Categories or Sectors.

The study found a statistical significance [F(4) = 3.62, *p* < 0.05] indicating that if people believed their institution’s methods for reporting rule-breaking (such as anonymous hotlines or online forms) were effective, they were also more likely to perceive the U.S. DURC policy as effective in reducing risks. This suggests that when institutions have strong systems in place to catch and report non-compliance, respondents may have greater confidence in the DURC policy’s ability to manage risks. Similarly, the results revealed a strong connection [F(4) = 4.65, *p* < 0.1] between perceptions of the effectiveness of these reporting methods and opinions on the U.S. ePPP policy’s effectiveness at reducing risks. However, it is important to consider an alternative explanation: individuals who believe that one system works well may also be inclined to think that other systems are effective, reflecting a general optimism (or pessimism) about the efficacy of systems in general. In this light, the observed relationship might reflect a broader confidence (or lack thereof) in institutional and national policies rather than the specific impact of reporting systems alone (See Table 8).

**TABLE 8:** Non-Compliance Reporting Mechanisms and Effectiveness of the DURC and U.S. ePPP Policies.

	Effectiveness of the institution’s non-compliance reporting mechanism to identify possible issues with DURC and ePPP experiments or oversight? (Question #16)					df^	Levene	F	ηp2
	Extremely effective M (SD)	Very effective M (SD)	Moderately effective M (SD)	Slightly effective M (SD)	Not effective at all M (SD)				
Effectiveness of DURC at reducing risk (Question #55)	3.21^a^ (±1.06)	2.81 (±0.89)	2.74 (±0.76)	2.2^a^ (±1.03)	2.00 (±1.41)	175	2.09	3.62*	0.08
Effectiveness of ePPP at reducing risk (Question #57)	3.06^ab^ (±1.03)	2.56 (±0.96)	2.70 (±0.82)	2.00^a^ (±0.67)	1.33^b^ (±0.58)	141	1.44	4.65**	0.12

Notes. *p < .05, **p < .01, ***p < .001. Groups sharing the same symbol (a, b) are significantly different from each other at the *0.05* significance level according to the Tukey HSD, test. ^ Degree Freedom (*df*) is the same for Levene’s test and F test.

## 4 Discussion

The findings of this study provide new empirical data about current DURC and ePPP oversight in the United States, noting significant differences in institutional practices, perceptions, and challenges. These findings demonstrate the complexity of managing biosafety and biosecurity risks and reveal how different accountability infrastructures in diverse institutional settings reveal critical differences in management practices, financial support, and the impact of oversight policies across various organizations and Sectors.

The analysis revealed significant differences in institutional practices related to DURC and ePPP activities, with Government institutions being more likely to conduct DURC compared to other institutions. The size of an institution’s biosafety/biosecurity team was positively correlated with the amount of research falling under the DURC policy, U.S. ePPP policy and the effectiveness of non-compliance reporting mechanisms (Survey Question #18, Question #32 and Question #16, respectively).

**FIGURE 1:**
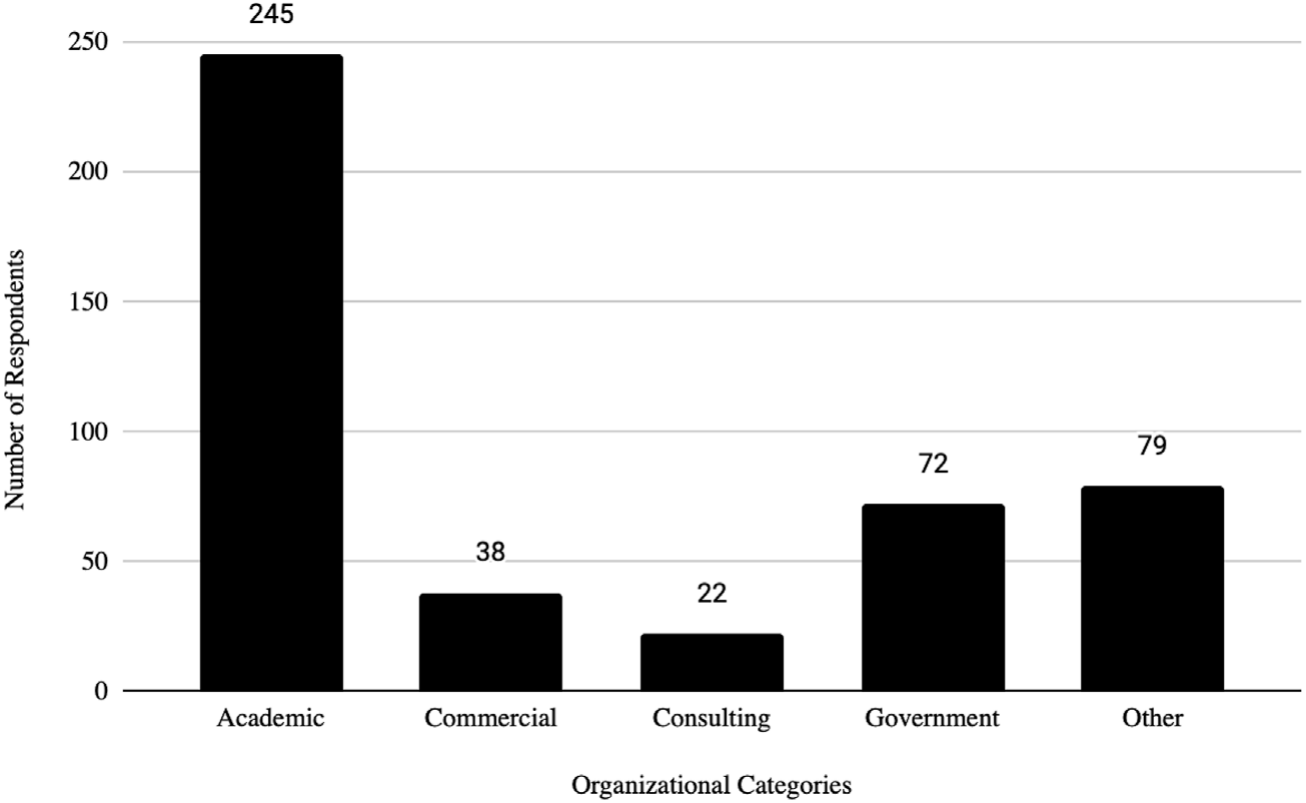
Organizational category of respondents.

Results indicated that Public and Private Non-Profit institutions are more likely to review experiments beyond the DURC policy compared to Private For-Profit. This is also true for institutions with larger biosafety/biosecurity teams. While there was no significant difference in the rate of ePPP research among different institution types, having larger biosafety/biosecurity teams was associated with institutions conducting more ePPP research. The effectiveness of non-compliance reporting mechanisms varied significantly across Organizational Categories (i.e., Academic, Commercial, Consulting, Government, Other; see Figure 1), with larger biosafety/biosecurity teams reporting having more effective mechanisms. These mechanisms could include anonymous whistleblower hotlines, secure online reporting forms, direct reporting to oversight committees (such as Institutional Biosafety Committees), confidential internal audits, designated ombuds personnel for ethical concerns, incident reporting systems required by specific guidelines, and integration with research integrity offices. Additionally, institutions with effective non-compliance reporting mechanisms tended to rate the DURC and ePPP policies as more effective in lowering risks than those reporting less-effective reporting mechanisms.

Opinions on the impact of the DURC and ePPP policies varied across different Organizational Categories and Sectors, suggesting that where one works may influence perceptions of these policies’ effectiveness. However, it’s important to note that other factors, such as the amount of DURC or ePPP research conducted within an institution, could also play a role in shaping these views. Regarding perceived financial support for DURC oversight, the study found no significant differences in funding across different Organizational Categories, indicating that similar levels of perceived financial support were provided regardless of the type of organization. However, a significant difference emerged between the Public and Private Sectors, with Public institutions receiving more perceived financial support than Private ones for DURC oversight. While the study identified this significant difference, it was unable to determine the exact amount of the disparity.

**FIGURE 2:**
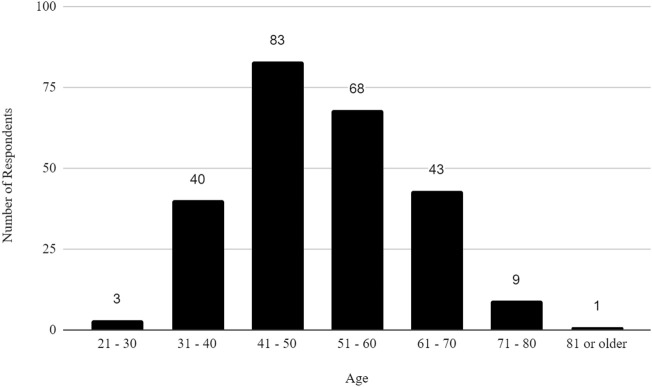
Age of respondents.

**FIGURE 3:**
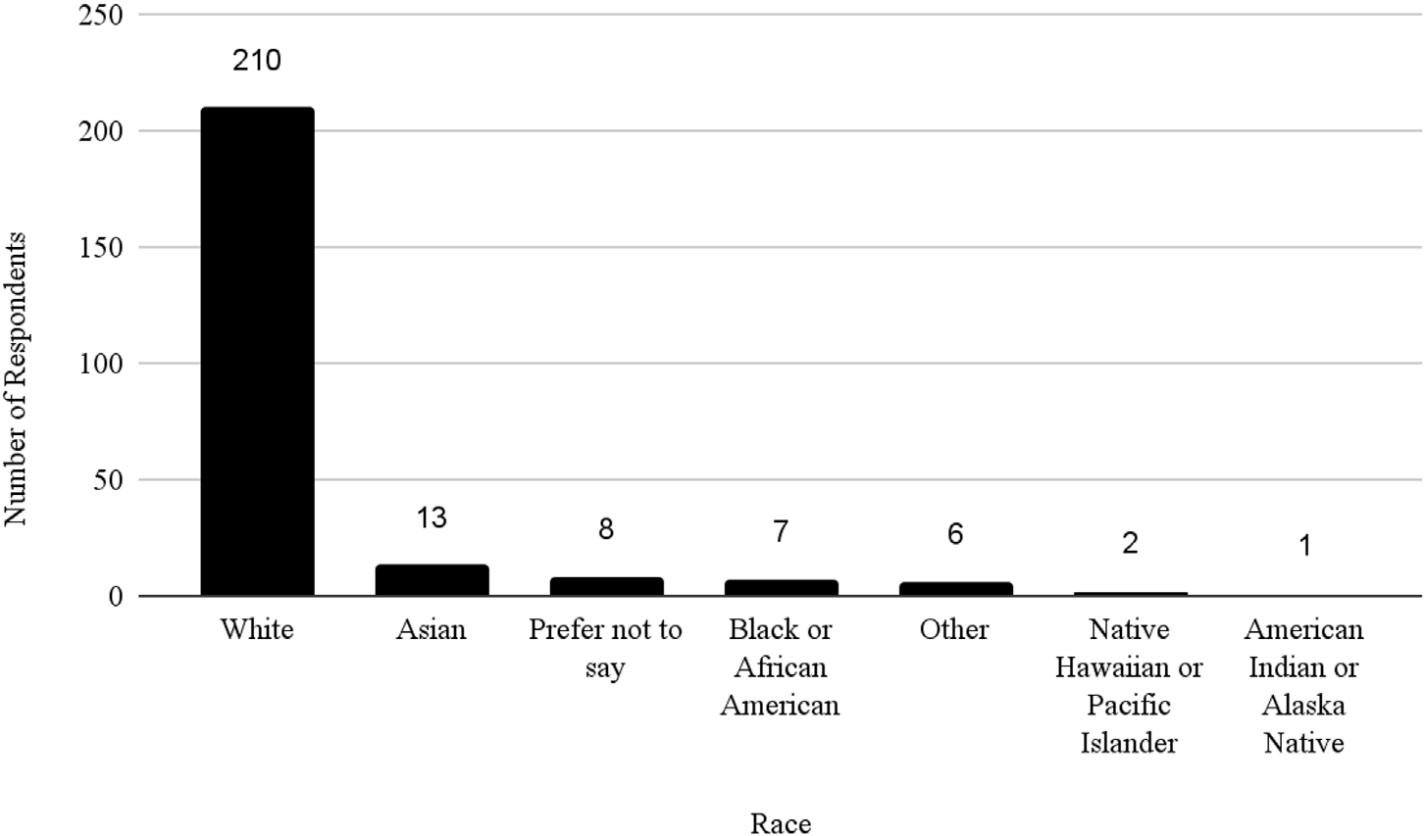
Race of respondents.

**FIGURE 4:**
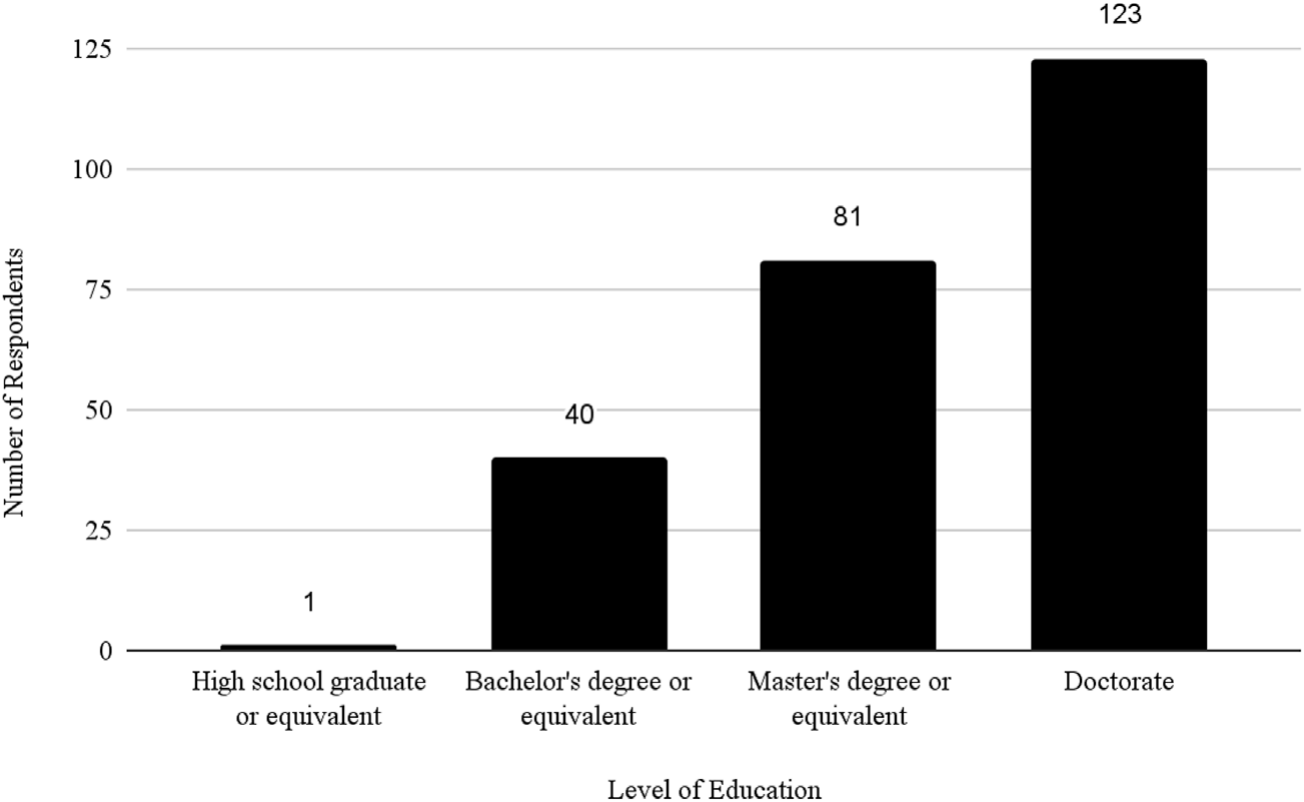
Level of education of respondents.

**FIGURE 5:**
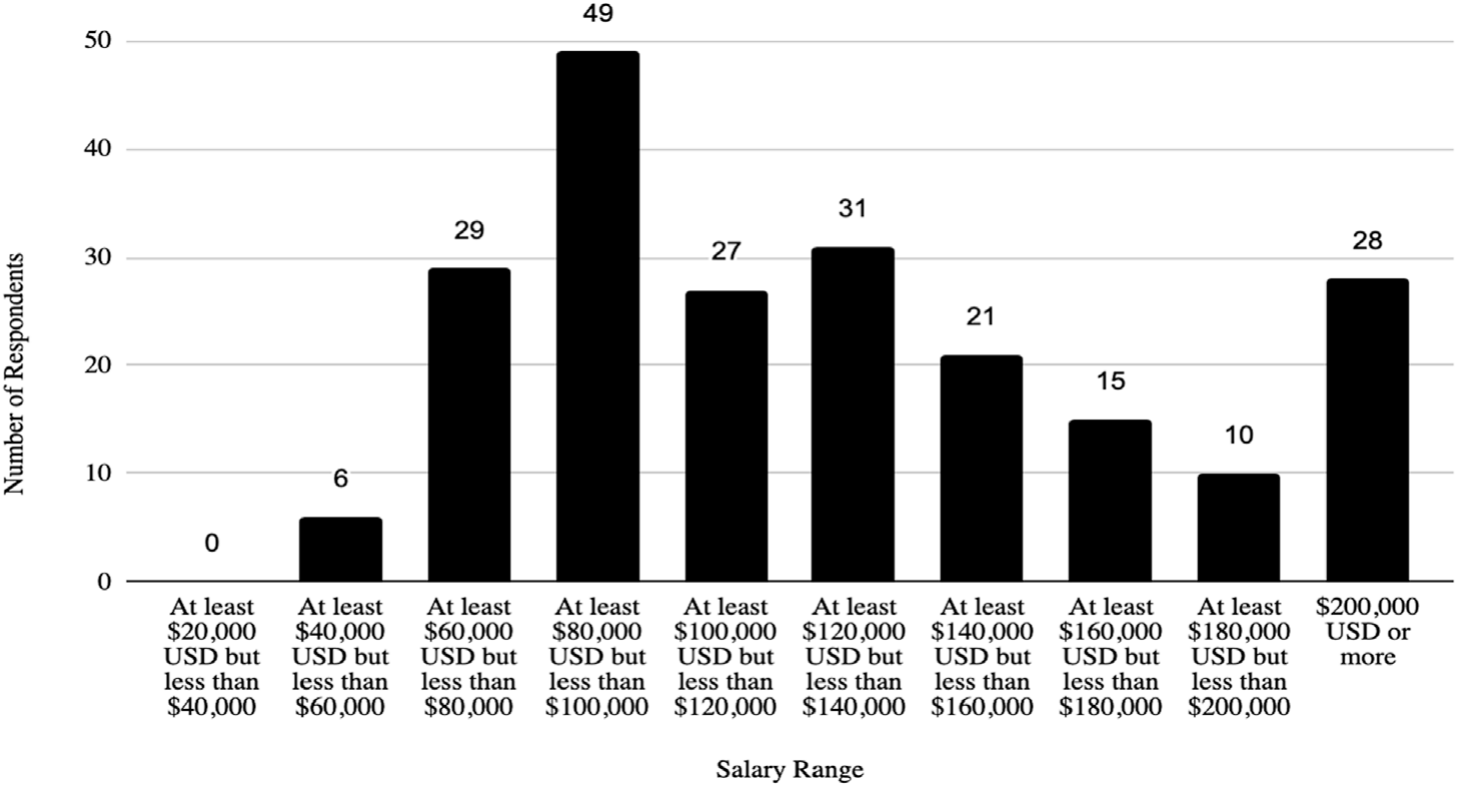
Salaries of respondents.

In addition, the survey provides an insight into the demographic backgrounds of respondents, which is important to consider when developing policies to help improve biosafety and biosecurity in the future. The survey revealed that a significant number of professionals have extensive experience in the field. Sixty-three respondents (12.1%) reported having more than 20 years of experience, indicating a wealth of knowledge and expertise in the field. Those with at least 15–20 years of experience accounted for 7.9%, followed by respondents with 10–15 years (11%), and those with 5–10 years (8.9%). This distribution shows a strong presence of seasoned professionals who have long standing experience implementing FSAP, DURC, and ePPP policies, with over 20% of respondents having more than 15 years of experience. This depth of experience is crucial for maintaining high standards in biosafety and biosecurity practices, as these individuals likely play key roles in mentoring newer professionals and shaping institutional policies. Understanding the age of respondents can also help with planning efforts to ensure there is capacity to fill jobs when older biosafety and biosecurity professionals retire. (see Table 1; Figures 2–5 for highlighted demographics of respondents).

### 4.1 Institutional variability in DURC and ePPP research

The research team discovered that out of the 126 people who said they were engaged in DURC, 34 worked for Private organizations. Notably, out of 64 respondents who reported conducting research on ePPP experiments, 23 worked for Private companies. This information is significant due to the attention devoted to Academic institutions conducting ePPP research, with little transparency on what ePPP research is occurring in the Private Sector. Also, of the 173 people who reported that their institutions conducted FSAP, 128 said their institutions conducted DURC, and 64 respondents (from at least 49 unique institutions) stated that their institutions performed ePPP experiments. This is especially interesting since only 3 institutions submitted their ePPP research to the Department of Health and Human Services for review (Office of the Assistant Secretary for Preparedness and Response, 2017). This finding could be due to various reasons, (e.g., the respondents are interpreting the definition of ePPP research very differently than the Department of Health and Human Services does, that the vast majority of ePPP research is not subject to P3CO review (presumably because it is not federally funded), or that DHHS is missing the vast majority of ePPP research. Our findings cannot determine what is leading to this discrepancy, but it is worth further examination in a future study.

The fact that more people reported that their institutions conducted FSAP-related activities could be influenced by the longevity of FSAP regulations, which have been in place since 2002, compared to the more recent implementation of DURC policy oversight in 2014 and ePPP policy oversight in 2017. However, it’s also important to consider that FSAP regulations, which involve a specific list of agents, represent an older and more established approach to biosecurity compliance. In contrast, DURC and ePPP policies, which involve both a list of agents and considerations of experimental outcomes within a funding context, may represent a newer and less familiar oversight process. This could contribute to a lower level of familiarity with DURC and ePPP policies compared to FSAP regulations. It should be noted that all DURC experiments involve a subset of FSAP agents, which may partly explain why FSAP is more frequently reported, unless institutions are conducting DURC research not formally covered by the U.S. DURC policies.

While most results did not vary for ePPP, one of the more striking findings from the survey is the significant variability in how organizations conduct and manage DURC (see Table 9 for a typology of factors influencing compliance in biosafety and biosecurity based upon this research). Government institutions were found to be more likely to engage in DURC compared to other Organizational Categories, which may be attributed to the amount of resources at the institution. For example, the positive correlation between the size of an institution’s biosafety/biosecurity team and the likelihood of conducting DURC research suggests that biosafety groups with more than one person are better equipped to handle the complexities and demands of such high-risk research. Based on our analysis, the optimal team size of respondents is four. This indicates that institutions with larger biosafety/biosecurity teams may have the resources and specialized expertise necessary to conduct thorough reviews and implement effective oversight. However, it is important to note that while larger teams are associated with increased research activities and more effective non-compliance reporting mechanisms, the overall effectiveness of biosafety and biosecurity practices also depends on other factors such as the quality of training, financial support, and the institution’s commitment to compliance and risk management.

**TABLE 9:** Organizational Attributes, Compliance Conditions, and Research Gaps in Biosafety and Biosecurity for DURC and PPP. This table presents a typology of factors influencing compliance in biosafety and biosecurity as it relates to this research. It categorizes key organizational characteristics, conditions affecting compliance, and open research questions, providing a multi-dimensional framework to understand the complexities of managing DURC and ePPP. This typology aims to guide future research and policy development by highlighting critical areas for improvement and investigation. Note: This typology is based on articles by McNie et al. that presents a multi-dimensional framework for categorizing different aspects of scientific research and stakeholder engagement. (McNie et al., 2015; McNie et al., 2016).

Organizational characteristics/Attributes	Conditions affecting compliance	Open questions/Gaps in research
Type of Institution	Government, Public, Private Non-Profit, Private For-Profit	How do different institution types vary in their approach to DURC and ePPP compliance?
Size of Biosafety/Biosecurity Team	Number of dedicated personnel, Allocation of responsibilities	What is the optimal team size for effective DURC and ePPP oversight?
Financial Resources	Level of funding for biosafety/biosecurity, Availability of grants and external support	How does funding variability impact compliance effectiveness?
Compliance Mechanisms	Effectiveness of non-compliance reporting systems, Pathways for reporting non-compliance	What are the best practices for non-compliance reporting in diverse institutional contexts?
Risk Mitigation Strategies	Day-to-day practices for risk assessment and management, Informal practices and unwritten rules	How do informal practices complement formal risk mitigation strategies?
Training and Education	Quality and frequency of DURC and ePPP training, Updates to training to keep pace with advances in biotechnology	What are the most effective training methods for biosafety and biosecurity?
Adherence to Policies	Implementation of U.S. Government Policy on DURC, Compliance with the Health and Human Services Enhanced Potential Pandemic Pathogens Framework	How can policy adherence be improved across various institution types?
Policy Impact	Perceived difficulty and impact of DURC and ePPP policies on research, Institutional reviews and assessments of DURC beyond minimum requirements	What are the unintended consequences of current DURC and ePPP policies on research productivity?
Ethical Frameworks	Integration of ethical guidelines in policy decisions, Handling of ethical dilemmas in research practices	How can ethical frameworks be better integrated into biosafety and biosecurity policies?
Stakeholder Engagement	Involvement of diverse perspectives in policy formulation, Transparency with internal and external stakeholders	What are the best practices for engaging stakeholders in biosafety and biosecurity?
Leadership and Governance	Role of senior leadership in fostering a culture of compliance, Governance structures for oversight and accountability	How does leadership influence compliance culture within institutions?
Collaboration and Coordination	Cross-departmental collaboration within institutions, Partnerships with external organizations and consultants	What are the benefits and challenges of cross-departmental and external collaboration in biosafety and biosecurity?
International Cooperation	Global regulatory frameworks and norms, Collaboration and transparency in international research	How can international cooperation be strengthened to enhance global biosafety and biosecurity?
Public Perception and Culture	Influence of popular culture on biosafety perceptions, Public trust and support for biosafety policies	How does public perception impact the implementation of biosafety policies?
Resource Limitations	Staffing and funding constraints, Access to necessary infrastructure and technology	What innovative solutions can address resource limitations in biosafety and biosecurity?
Regulatory Complexity	Conflicting or unclear guidelines, Administrative burdens and paperwork	How can regulatory frameworks be streamlined to reduce complexity and improve compliance?
Research Freedom vs. Safety	Balancing scientific advancement with risk management, Impact of policies on research timelines and flexibility	How can policies balance the need for scientific innovation with biosafety and biosecurity concerns?
Case Studies and Learning	Lessons learned from compliance failures, Examples of effective risk mitigation	What can be learned from past compliance failures to improve future practices?

The government’s vested interest in national security and public health likely contributes to its greater engagement in DURC. Government institutions also have access to extensive funding and specialized facilities, and they benefit from robust regulatory oversight, which could make them more capable of managing DURC effectively. In contrast, Private Sectors may avoid DURC due to concerns about public perception, liability, and the lack of immediate commercial benefits, which further positions Government institutions as more suitable for conducting such research.

### 4.2 Compliance and oversight practices

**FIGURE 6:**
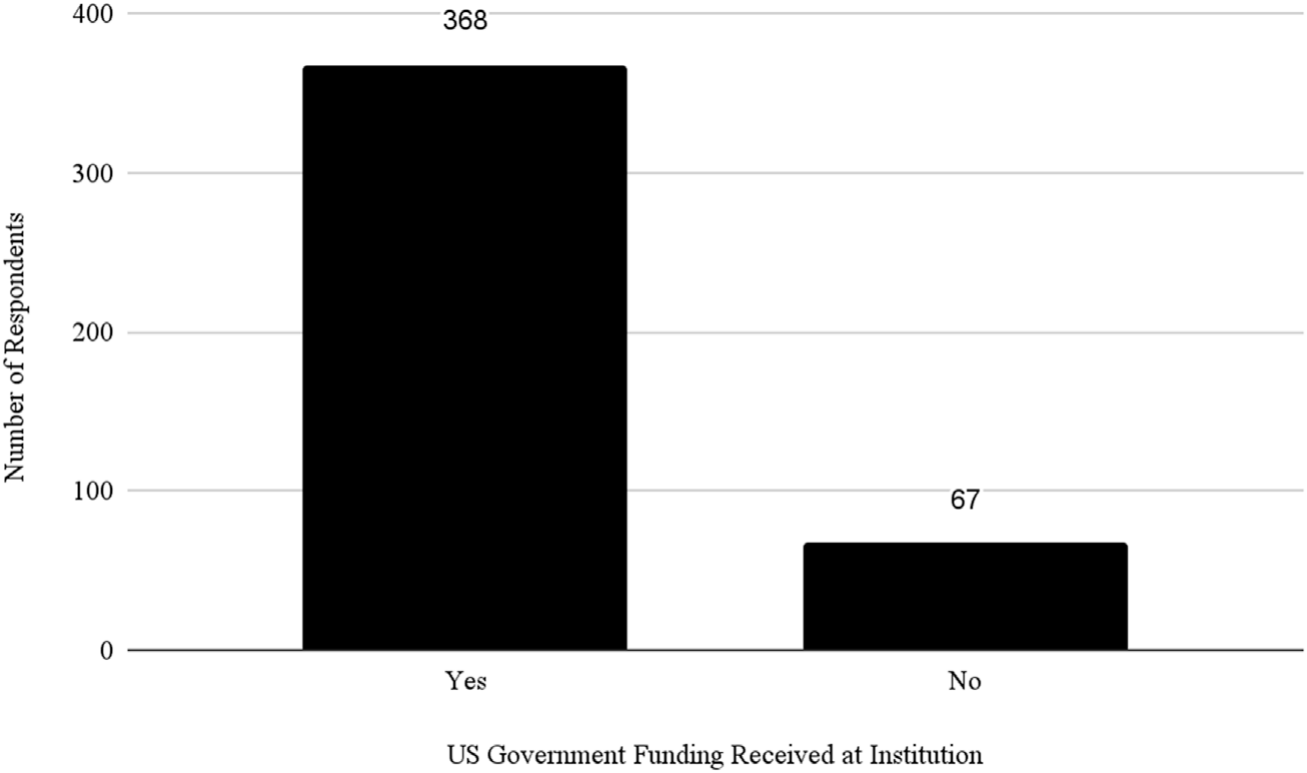
Number of institutions receiving U.S. Government funding.

Almost 50% of respondents said they worked at a Public institution and 71% of all respondents indicated that they received federal funding at their institution (see Figure 6). However, the study revealed significant differences in how institutions review and manage DURC and ePPP experiments. Public and Private Non-Profit institutions are more likely to review experiments beyond the scope of the DURC policy compared to Private For-Profit institutions. This could be due to the differing priorities and resource allocations between Non-Profit and For-Profit Sectors. To understand this better, it would be necessary to consider an organization’s risk management program, as well as their biosafety and biosecurity infrastructure and culture (Huising and Silbey, 2021; Silbey, 2016).

The effectiveness of non-compliance reporting mechanisms also varies significantly across institutions, with respondents indicating that larger biosafety/biosecurity teams demonstrate more effective compliance and reporting mechanisms. This acknowledges the importance of adequate staffing and resources to ensure robust oversight and compliance. The importance of adequate staffing in high-risk research facilities is described in a (National Research Council, 2009) report:

“While the challenges of sustainable funding for scientific research go far beyond select agent research and this report, the implications are more troubling in the case of select agent research. It is not acceptable, either for the institution or for safety and security, to diminish appropriate and necessary risk-based security procedures and resources, regardless of the availability of funding for the facility. Host institutions, having to provide the difference, may choose to reduce their cost by understaffing the facility, hiring external contractors where a third party takes responsibility for key functions, or diverting funds planned to support scientific research to pay for security responsibilities. These are not sustainable solutions and raise risks” (National Research Council, 2009, 132–133).

Respondents that rated their non-compliance reporting mechanisms as very effective also tended to rate the DURC and ePPP policies as more effective at lowering risks (see Table 8). This correlation highlights the critical role of internal compliance mechanisms in reinforcing external regulatory frameworks (Huising and Silbey, 2021).

### 4.3 Perception of financial support and policy impact

Significant differences were observed in the perception of financial support for DURC oversight across Public, Private For-Profit, and Private Non-Profit institutions. Public institutions reported higher levels of perceived financial support, which may reflect their access to more government funding and grants than private institutions. However, this disparity may also demonstrate the need for equitable resource allocation to ensure all institutions can effectively manage biosafety and biosecurity oversight for DURC. Additionally, it may be beneficial to assess an institution’s biosafety and biosecurity capacity before approving funding for a DURC project to ensure adequate measures are in place.

The perceived impact of the DURC policy on research varied significantly among organization types. Public institutions, which often face more stringent compliance requirements and public scrutiny, may experience greater administrative burdens and public relations challenges, which may impact research timelines and flexibility. Conversely, Private For-Profit institutions, which are not subject to ePPP policies (if they do not receive federal funding), may experience fewer regulatory hurdles, allowing for more agile research processes. Anecdotally it is important to understand that compliance with DURC and ePPP policies for those receiving federal funding is an unfunded mandate, and increased funding for institutional biosafety and biosecurity as part of funded DURC and ePPP research projects could improve compliance. These differences demonstrate the need for tailored policy approaches that consider the unique challenges faced by different types of organizations.

### 4.4 Challenges in policy implementation

Despite the established policies and guidelines, institutions face significant challenges in implementing DURC and ePPP policies. The difficulty of managing FSAP varies significantly among Organizational Categories, with Consulting and Commercial institutions perceiving increased challenges. Financial support for managing these policies also varies across institution types, with FSAP receiving the most financial support, followed by DURC, and ePPP receiving the least. Academic and Other institutions report significant differences in financial support for FSAP, DURC, and ePPP policies, indicating potential gaps in resource allocation that could hinder effective oversight.

Respondents indicated that the quality of the DURC training at their institution was somewhat good, while ePPP training was rated as neither good nor bad. Notably, no one reported the quality of DURC training as extremely bad. The effectiveness of how each workplace revises DURC and ePPP training to keep pace with advances in biotechnology and life sciences ranged from slightly to moderately well. This lack of excellence in training quality may be due to resource constraints, such as funding, time, and personnel, which impact the frequency and depth of training revisions. Additionally, the rapid developments in science, technology, and policy may make it challenging to continuously update training programs.

While not directly derived from the survey responses, strengthening governance frameworks to manage biosafety and biosecurity risks is crucial for maintaining the integrity of biological research. Ensuring that research involving high-risk pathogens is conducted under strict safety protocols will help reduce the likelihood of accidental releases or malicious use. Having a transparent, risk-based approach will help build public trust in scientific research and its regulatory bodies (Imperiale and Casadevall, 2018; U.S. Government Accountability Office, 2023). Incorporating ethical frameworks into policy decisions could help ensure that high-risk research is conducted under principles that prioritize human, animal, and environmental safety (Evans et al., 2015).

### 4.5 Implications for policy and practice

The findings of this study have several implications for policymakers, biosafety and biosecurity professionals, institutional leaders, members of the public, and others. The survey results indicate a need for tailored strategies that consider the unique challenges and resource limitations of different types of institutions. Policies should be designed to provide equitable support and resources, particularly for smaller institutions or those with fewer biosafety/biosecurity personnel.

The private sector’s high level of involvement in ePPP and DURC research, coupled with the lack of specific oversight for private institutions that do not receive federal funding, emphasizes the need for stronger regulatory frameworks and more robust oversight mechanisms in this sector. Enhancing the effectiveness of non-compliance reporting mechanisms, which are critical components of DURC and P3CO frameworks, should be a priority. Institutions with robust reporting mechanisms not only reported better compliance with existing policies but also perceived those policies as more effective in mitigating risks. Investment in the development and maintenance of these mechanisms should be a priority.

Furthermore, educating the public about the realities of biosafety and biosecurity risks and the measures in place to mitigate them is crucial for building trust and ensuring public support for necessary policy changes (Lentzos et al., 2020). By fostering informed public discourse, scientists and institutional biosafety officers could counteract misinformation and fear, creating a more resilient and knowledgeable society that supports robust biosafety and biosecurity measures.

### 4.6 Future research directions

This study lays the groundwork for future qualitative and quantitative research to further explore the practices, perceptions, and challenges related to high-risk biological research governance. Conducting interviews with biosafety and biosecurity professionals will provide richer insights into the nuances of policy implementation and the lived experiences of those responsible for managing these activities. Additionally, longitudinal studies can track changes in institutional practices and perceptions over time, providing valuable data to inform the ongoing evolution of biosafety and biosecurity policies. Understanding the long-term impacts of these policies on scientific research and public health will help with creating more dynamic and responsive oversight frameworks as science and technology develop.

Several data gaps limit the comprehensiveness of our findings. The study excluded responses from international participants, as the grant was to research only people in the United States, thus lacking a global perspective. Incomplete responses and significant portions of unspecified demographic data restrict the reliability and depth of the analysis based upon gender, age, years of experience, and so on. Similarly, the broad categorization of institutions may overlook nuanced differences within these categories. The small number of respondents from specific categories could further limit the accuracy and representatives of our findings. Additionally, future studies could improve by considering comparisons within the same institution and exploring the possibility of allowing multiple respondents from each institution, and also including queries that ask respondents about the time periods related to their responses. This approach may help capture more detailed and representative data. The curious discrepancy between the amount of ePPP research reported by our respondents and that documented by the U.S. Department of Health and Human Services is also worthy of additional research in a future study. We could also ask more fine-grained questions about the number and process for DURC reviews and the management of FSAP regulations, and how institutions handle noncompliance, to better understand how different organizations encounter and manage challenges across these different policies and regulations.

Comparative studies examining DURC and ePPP are scarce (Gaudioso and Zemlo, 2007; Sarwar et al., 2019; MacIntyre et al., 2020; Cadari et al., 2021; Vinke et al., 2022), with no known longitudinal analyses assessing the effectiveness of these policies over time. The integration of interdisciplinary approaches and stakeholder perspectives on biosafety and biosecurity is also limited; having these could provide a more holistic understanding of biosafety and biosecurity management.

To address these gaps, future research should focus on comprehensive and consistent data collection, including incentivizing respondents to complete survey questions. Expanding the scope of surveys and interviews to include international respondents would offer valuable comparative insights for the global community. By addressing these data and literature gaps, future research could provide more detailed and actionable plans for the oversight of DURC and ePPP research worldwide, ultimately contributing to a safer and more secure world. Understanding international approaches and different kinds of accountability infrastructures for biosafety and biosecurity regarding DURC and ePPP research is critical because we live in an interconnected world where pathogens do not recognize national borders. What occurs in one part of the world can have significant repercussions globally. By learning from international practices and policies, we can develop more robust and effective strategies to mitigate risks and improve biosafety and biosecurity around the world.

**FIGURE 7:**
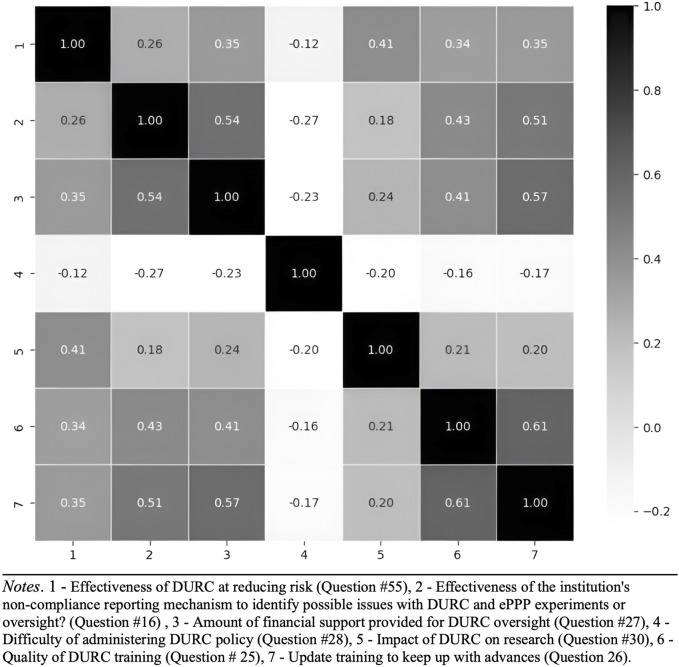
Correlation heatmap of DURC policy effectiveness and other factors.

**FIGURE 8:**
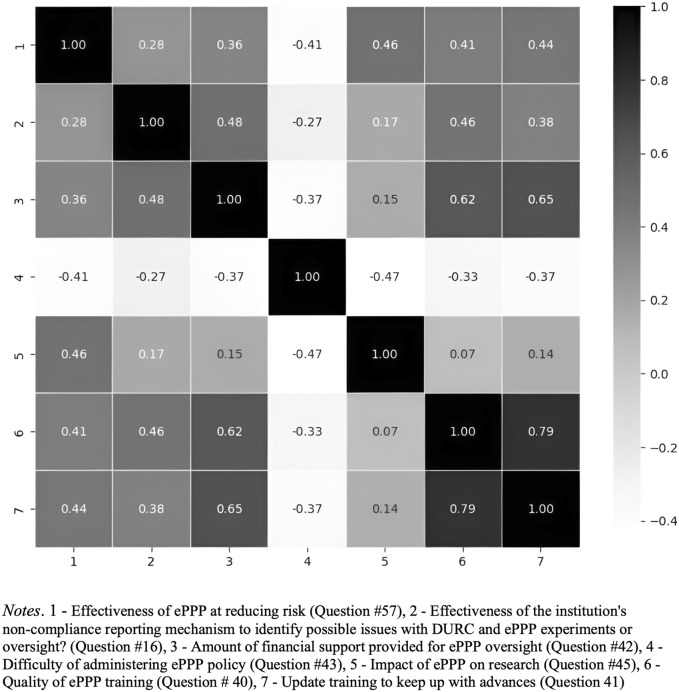
Correlation heatmap of ePPP policy effectiveness and Other Factors.

Based on the findings from the correlation heatmap (Figures 7, 8), which reveals relationships among various factors related to DURC and ePPP effectiveness, future research is recommended to investigate the interactions between financial support, perceived difficulty, impact assessment, and training quality in relation to the effectiveness of DURC/ePPP policies and the institutional non-compliance mechanism. Examining these relationships will provide insights into potential drivers of policy effectiveness and offer insights to improve these policies.

## 5 Conclusion

This study identifies critical gaps in biosafety and biosecurity practices, such as the significant variability in institutional practices related to DURC and ePPP management, the insufficient financial support and resource allocation perceived for effective oversight, particularly in smaller institutions, and the disparities in the perceived effectiveness of non-compliance reporting mechanisms. Additionally, the study highlights the challenges in maintaining adequate staffing levels, the uneven quality of training programs, and the lack of standardized procedures across different sectors, all of which contribute to the inconsistent application of biosafety and biosecurity measures across U.S. institutions. Government and public institutions are more likely to conduct DURC research, and larger biosafety teams correlated with increased research activity and more effective non-compliance reporting mechanisms. Financial support and policy implementation challenges vary significantly across institutional sectors. Private institutions may be less involved in DURC because of the lack of immediate commercial benefits, resource constraints, and concerns about public perception and liability issues, but also because privately funded research at these institutions is not subject to the DURC policy, meaning that DURC research might be conducted without being flagged or identified.

Based upon the author’s experiences, training for DURC and ePPP varies by institution. While some institutions develop their own training programs, others may utilize resources from organizations like the American Biological Safety Association (ABSA), which offers training modules and certification programs. However, the current DURC and ePPP policies do not specify detailed training requirements, only stating that personnel must be trained. This vagueness presents an opportunity to improve policies by defining clear, measurable training standards and providing funding to support compliance. Improved policies could include mandatory, standardized training programs with regular updates, assessments to ensure comprehension and effectiveness, and dedicated resources from institutions and financial incentives from the government to help institutions meet these requirements.

In contrast, FSAP training requirements are more rigid by law, specifying detailed criteria, mandatory initial and annual refresher training, and insider threat awareness briefings, with strict record-keeping for compliance (Federal Select Agent Program, 2024). Standardizing DURC and ePPP training to similar levels of rigor could enhance the overall effectiveness and reliability of biosafety and biosecurity practices.

This research underscores the need for improved regulatory approaches and more equitable resource distribution strategies to manage high-risk biological research effectively. Strengthening internal compliance mechanisms, improving training quality, and fostering international cooperation are vital for improving biosafety and biosecurity oversight. Future research should focus on developing more comprehensive and consistent data collection, including international perspectives and longitudinal studies, to provide actionable insights for policy improvements.

By addressing these issues, institutions and policymakers can create a safer and more innovative research environment, ensure robust management of DURC and ePPP experiments and foster public trust in scientific research.

## Data Availability

The original contributions presented in the study are publicly available. This data can be found here: https://doi.org/10.7910/DVN/UDYIF8.
