# Supplementary material for: Bridging biosafety and biosecurity gaps: DURC and ePPP policy insights from U.S. institutions
Source: Front Bioeng Biotechnol. 2024 Sep 25;12:1476527. doi: 10.3389/fbioe.2024.1476527 (PMC11467424; doi:10.3389/fbioe.2024.1476527)
Supplement: Supplementary file 1 [file DataSheet1.DOCX]

**Supplement A. Survey Questions for Biosafety and Biosecurity Professionals.**

This supplement lists the comprehensive survey questions designed to gather insights from respondents on their practices, responsibilities, and perceptions related to DURC and ePPP. These questions aim to assess institutional compliance, ethical considerations, and the effectiveness of current policies, providing valuable data to inform improvements in biosafety and biosecurity oversight.

1. **Are you currently employed in a position with biosafety responsibilities?** Yes; No; Not applicable
2. **If No, are you currently a student, retiree, or unemployed?** Yes; No; Not applicable
3. **Are you a consultant in a position with biosafety responsibilities?** Yes; No; Not applicable
4. **Are you the Biological Safety Officer (BSO) where you work?** Yes; No; Not applicable
5. **Do you have an Institutional Biosafety Committee (IBC) where you work?** Yes; No; Not applicable
6. **Outsourced Is the Institutional Biosafety Committee (IBC) outsourced?** Yes; No; Not applicable
7. **Do you feel that you are effectively able to implement biosafety / biosecurity oversight where you work?** Yes; No; Not applicable
8. **Would you please elaborate on why you are unable to effectively implement biosafety / biosecurity oversight where you work?**
9. **Are you in a role with biosecurity responsibilities?** Yes; No; Not applicable
10. **Approximately how much of your time is spent on biosafety and how much is spent on biosecurity?** Primarily biosafety (~100% biosafety / ~0% biosecurity), Mostly biosafety with a little biosecurity (~75% biosafety / ~25% biosecurity), About the same amount of biosafety and biosecurity (~50% biosafety / ~50% biosecurity), Mostly biosecurity with a little biosafety (~75% biosecurity / ~25% biosafety), Primarily biosecurity (~100% biosecurity / ~0% biosafety), I don't know, Not applicable
11. **Does your workplace receive funding from the U.S. Government?** Yes; No; Not applicable
12. **Which category best describes the institution where you work?** Clinical or Diagnostic Testing; Commercial; Consulting Company; Contract Research Organization Government - Federal, State, City, Tribal; Government Owned / Privately Operated; Pharmaceutical; Undergraduate University / Primary Teaching Institution; Master's College or University (Carnegie M1-M3); R1 Doctoral University – Very High Research Activity; R2 Doctoral University – High Research Activity; Other; Not applicable
13. **How would you characterize the institution where you work?** Private - For-Profit; Private - Non-Profit; Public; I don't know; Not applicable
14. **Which of the following biosafety facilities are at your institution? (Please select all that apply.)** BSL-1 or equivalent; BSL-2 or equivalent; BSL-3 or equivalent; BSL-4 or equivalent; Not applicable
15. **How many biosafety / biosecurity practitioners work at your institution?** (If an employee is partially employed as a biosafety / biosecurity practitioner, please round up to a whole number.) None; 1; 2; 3; 4; 5 or more; Not applicable; I don't know
16. **How effective is your institution's non-compliance reporting mechanisms to identify possible issues with Dual Use Research of Concern (DURC) and/or Enhanced Potential Pandemic Pathogen (ePPP) experiments or oversight?** Not effective at all; Slightly effective; Moderately effective; Very effective; Extremely effective; My institution does not have a non-compliance reporting mechanism for DURC and/or ePPP;\ I don't know; Not applicable
17. **Do you have a pathway for reporting non-compliance with oversight of Dual Use Research of Concern (DURC) and/or Enhanced Potential Pandemic Pathogens (ePPP) experiments where you work?** Yes; No; I don't know; Not applicable
18. **Does your workplace conduct research that is subject to the U.S. Government Policy on Dual Use Research of Concern?** Yes; No; I don't know; Not applicable
19. **Does your workplace outsource research that is subject to the U.S. Government Policy on Dual Use Research of Concern?** Yes; No; I don't know; Not applicable
20. **How does your workplace determine if research is covered by the U.S. Government Policy on Dual Use Research of Concern?** Please check all that apply. Review by laboratory personnel; Review by principal investigator; Review by biosafety officer; Review by consultant; Review by IBC compliance coordinator; Review by Institutional Biosafety Committee; Review by senior leadership; Other; Not reviewed; I don't know; Not applicable
21. **Does your workplace conduct reviews for Dual Use Research of Concern (DURC) for experiments that are not covered by the U.S. Government Policy on Dual Use Research of Concern?** (For example, does your workplace conduct DURC assessments for experiments that do not include the 15 agents and seven experimental effects that are identified in the Policy?) Yes; No; I don't know; Not applicable
22. **If your workplace conducts reviews for Dual Use Research of Concern (DURC), what kind of research is reviewed? Please check all that apply. [Note: The current U.S. Policy applies to only Avian influenza virus (highly pathogenic); Bacillus anthracis; Botulinum neurotoxin; Burkholderia mallei; Burkholderia pseudomallei; Ebola virus; Foot-and-mouth disease virus; Francisella tularensis; Marburg virus; Reconstructed 1918 Influenza virus; Rinderpest virus; Toxin-producing strains of Clostridium botulinum; Variola major virus; Variola minor virus; and Yersinia pestis]** Agents listed in the U.S. Dual Use Research of Concern Policy (see list above); All research regardless of Biosafety Level (BSL); Research conducted at Biosafety Level (BSL) 1; Research conducted at Biosafety Level (BSL) 2; Research conducted at Biosafety Level (BSL) 3; Research conducted at Biosafety Level (BSL) 4; Self-Replicating Nucleic Acids; Select Agents and Toxins; Other; We do not conduct reviews for DURC at our institution; Not applicable
23. **Does your workplace have an Institutional Review Entity (IRE)?** Yes; No; I don't know; Not applicable
24. **Does your workplace train researchers about the risks associated with Dual Use Research of Concern (DURC)?** Yes; No; I don't know; Not applicable
25. **In your opinion, how is the quality of the Dual Use Research of Concern (DURC) training where you work?** Extremely bad; Somewhat bad; Neither good nor bad; Somewhat good; Extremely good; I don't know; Not applicable
26. **How well does your workplace update training to keep up with advances in biotechnology and the life sciences that can contribute to new risks involving Dual Use Research of Concern (DURC)?** Not well at all; Slightly well; Moderately well; Very well; Extremely well; I don't know; Not applicable
27. **How would you describe the amount of financial support provided for Dual Use Research of Concern (DURC) oversight where you work?** Poor; Fair; Good; Very good; Excellent; I don't know; Not applicable
28. **How difficult is the U.S. Government's Dual Use Research of Concern (DURC) policy to administer/manage where you work?** Extremely difficult; Somewhat difficult; Neither easy nor difficult; Somewhat easy; Extremely easy; I don't know; Not applicable
29. **If difficult, would you please elaborate on why it is difficult?**
30. **How would you describe the impact of the U.S. Government's Dual Use Research of Concern (DURC) policy on research where you work?** Extremely negative; Somewhat negative; Neither positive nor negative; Somewhat positive; Extremely positive; I don't know; Not applicable
31. **Would you please elaborate on the impact on research?**
32. **Does your workplace conduct research that may generate enhanced Potential Pandemic Pathogens (ePPP)?** Yes; No; I don't know; Not applicable
33. **Does your workplace outsource research that is subject to the U.S. Health and Human Services Enhanced Potential Pandemic Pathogens Framework?** Yes; No; I don't know; Not applicable
34. **Has any research at your workplace undergone a review under the U.S. Health and Human Services Enhanced Potential Pandemic Pathogens Framework?** Yes; No; I don't know; Not applicable
35. **If Yes, what was the outcome of that review?**
36. **How does your workplace determine if research is covered by the U.S. Health and Human Services Enhanced Potential Pandemic Pathogens Framework? Please select all that apply.** Review by laboratory personnel; Review by the principal investigator; Review by the biosafety officer; Review by consultant; Review by the IBC compliance coordinator; Review by the Institutional Biosafety Committee; Review by senior leadership; Other; Not reviewed; I don't know; Not applicable
37. **If your workplace conducts reviews for enhanced Potential Pandemic Pathogens (ePPP), what kind of research is reviewed? Please check all that apply. [Note: The current policy lists H5N1 influenza viruses and SARS-CoV-2 as examples.]** Influenza viruses; MERS viruses; SARS viruses; Respiratory / aerosol-borne pathogens; Risk Group 1 agents; Risk Group 2 agents; Risk Group 3 agents; Risk Group 4 agents; Other specific agents; I don't know; Not applicable
38. **How prepared do you feel you are in understanding the risks associated with enhanced Potential Pandemic Pathogens (ePPP)?** Extremely unprepared; Somewhat unprepared; Neither prepared nor unprepared; Somewhat prepared; Extremely prepared; I don't know; Not applicable
39. **Does your workplace train researchers about the risks associated with enhanced Potential Pandemic Pathogens (ePPP)?** Yes; No; I don't know; Not applicable
40. **How is the quality of the enhanced Potential Pandemic Pathogens (ePPP) training where you work?** Extremely bad; Somewhat bad; Neither good nor bad; Somewhat good; Extremely good; I don't know; Not applicable
41. **How well does your workplace update training to keep up with advances in biotechnology and the life sciences that can contribute to new risks involving enhanced Potential Pandemic Pathogens (ePPP)?** Not well at all; Slightly well; Moderately well; Very well; Extremely well; I don't know; Not applicable
42. **How would you describe the amount of financial support provided for enhanced Potential Pandemic Pathogen (ePPP) oversight where you work?** Poor; Fair; Good; Very good; Excellent; I don't know; Not applicable
43. **How difficult is the U.S. Government's enhanced Potential Pandemic Pathogen (ePPP) policy to administer/manage where you work?** Extremely difficult; Somewhat difficult; Neither easy nor difficult; Somewhat easy; Extremely easy; I don't know; Not applicable
44. **If difficulty, would you please elaborate on the difficulty?**
45. **How would you describe the impact of the U.S. Government's enhanced Potential Pandemic Pathogen (ePPP) policy on research where you work?** Extremely negative; Somewhat negative; Neither positive nor negative; Somewhat positive; Extremely positive; I don't know; Not applicable
46. **If negative, would you please elaborate on the impact on research?**
47. **Does your workplace use a subcontractor or subawardee to conduct Dual Use Research of Concern (DURC) and/or Enhanced Potential Pandemic Pathogen experiments (ePPP)?** Yes; No; I don't know; Not applicable
48. **Does your workplace conduct research governed by the Federal Select Agent and Toxin regulations?** Yes; No; I don't know; Not applicable
49. **Are you the Responsible Official (RO) or Alternate Responsible Official (ARO) for the Select Agent and Toxin program where you work?** Yes; No; I don't know; Not applicable
50. **How would you describe the amount of financial support provided for Select Agent and Toxin oversight where you work?** Poor; Fair; Good; Very good; Excellent; I don't know; Not applicable
51. **How difficult are the government's regulations for Select Agents and Toxins to manage where you work?** Extremely difficult; Somewhat difficult; Neither easy nor difficult; Somewhat easy; Extremely easy; I don't know; Not applicable
52. **If difficult, would you please elaborate on why it is difficult?**
53. **How would you describe the impact of the U.S. Select Agent and Toxins regulations on research where you work?** Extremely negative; Somewhat negative; Neither positive nor negative; Somewhat positive; Extremely positive; I don't know; Not applicable
54. **Would you please elaborate on the impact to research?**
55. **In your opinion, how effective is the U.S. policy on Dual Use Research of Concern (DURC) at reducing risks?** Not effective at all; Slightly effective; Moderately effective; Very effective; Extremely effective; I don't know
56. **Please provide any additional comments / feedback about how effective (or ineffective) the U.S. DURC policy is at reducing risk.**
57. **In your opinion, how effective is the oversight of enhanced Potential Pandemic Pathogen (ePPP) at reducing risks?** Not effective at all; Slightly effective; Moderately effective; Very effective; Extremely effective; I don't know
58. **Please provide any additional comments / feedback about how effective (or ineffective) the U.S. ePPP policy is at reducing risk.**
59. **Are you knowledgeable of the recent regulatory framework proposed by the Office of Science Policy Technology (FR Doc. 2023–18906 Filed 8–31–23; Request for Information; Potential Changes to the Policies for Oversight of Dual Use Research of Concern (DURC) and the Potential Pandemic Pathogen Care and Oversight (P3CO) Policy Framework)?** Yes; No; I don't know
60. **Background: The following demographic questions are being asked to better understand who is working in the field of biosafety and biosecurity. Would you like to provide demographic information about yourself?** Yes; No
61. **What is your gender?** Male; Female; Non-binary / third gender; Other; Prefer not to say
62. **What is your sexual orientation?** Heterosexual; Homosexual; Bisexual; Other; Prefer not to say
63. **What is your age?** 18 - 20; 21 - 30; 31 - 40; 41 - 50; 51 - 60; 61 - 70; 71 - 80; 81 or older; Prefer not to say
64. **What is your race?** White; Black or African American; American Indian or Alaska Native; Asian; Native Hawaiian or Pacific Islander; Other; Prefer not to say
65. **What is your ethnicity?** Hispanic or Latino or Spanish Origin; Not Hispanic or Latino or Spanish Origin; Other; Prefer not to say
66. **What is your highest level of education?** Less than high school graduate; High school graduate or equivalent; Associate's degree or equivalent; Bachelor's degree or equivalent; Master's degree or equivalent; Doctorate; Other; Prefer not to say
67. **What is your total accumulated years of experience performing biosafety / biosecurity - related duties?** Less than 1 year; At least 1 year but less than 5 years; At least 5 years but less than 10 years; At least 10 years but less than 15 years; At least 15 years but less than 20 years; More than 20 years; Other; Prefer not to say
68. **What is your average annual gross salary in U.S. dollars, rounded to the nearest $1,000? For example, if you make $84,243, your response would be $84,000. Note: Gross salary is the amount of pay before any deductions have been taken out of your wages.**
69. **Are you interested in participating in more in-depth surveys, interviews, and meetings regarding biosafety / biosecurity policy development and oversight?** Yes; No
70. **If you are interested in participating in more in-depth surveys, interviews, and meetings regarding biosafety / biosecurity policy development and oversight, would you please provide your first and last name and e-mail address in the text box below? Note: We will disaggregate responses to this question to ensure the survey is blinded. Answers will not be able to be attributed back to the source.** First Name; Last Name; E-mail
